# Machine learning identifies SLC6A14 as a novel biomarker promoting the proliferation and metastasis of pancreatic cancer via Wnt/β-catenin signaling

**DOI:** 10.1038/s41598-024-52646-8

**Published:** 2024-01-24

**Authors:** Cunshu Dang, Quan Bian, Fengbiao Wang, Han Wang, Zhipeng Liang

**Affiliations:** 1 Department of Hepatobiliary Gastrointestinal Surgery, Tianjin Fourth Central Hospital, No.1 Zhongshan Road, Tianjin, China; 2 Department of Plastic and Reconstructive Surgery, Tianjin Nankai Hospital, Tianjin, China; 3 Department of Otorhinolaryngology-Head and Neck Surgery, Tianjin Fourth Central Hospital, Tianjin, China

**Keywords:** Cancer, Cell biology

## Abstract

Pancreatic cancer (PC) has the poorest prognosis compared to other common cancers because of its aggressive nature, late detection, and resistance to systemic treatment. In this study, we aimed to identify novel biomarkers for PC patients and further explored their function in PC progression. We analyzed GSE62452 and GSE28735 datasets, identifying 35 differentially expressed genes (DEGs) between PC specimens and non-tumors. Based on 35 DEGs, we performed machine learning and identified eight diagnostic genes involved in PC progression. Then, we further screened three critical genes (CTSE, LAMC2 and SLC6A14) using three GEO datasets. A new diagnostic model was developed based on them and showed a strong predictive ability in screen PC specimens from non-tumor specimens in GEO, TCGA datasets and our cohorts. Then, clinical assays based on TCGA datasets indicated that the expression of LAMC2 and SLC6A14 was associated with advanced clinical stage and poor prognosis. The expressions of LAMC2 and SLC6A14, as well as the abundances of a variety of immune cells, exhibited a significant positive association with one another. Functionally, we confirmed that SLC6A14 was highly expressed in PC and its knockdown suppressed the proliferation, migration, invasion and EMT signal via regulating Wnt/β-catenin signaling pathway. Overall, our findings developed a novel diagnostic model for PC patients. SLC6A14 may promote PC progression via modulating Wnt/β-catenin signaling. This work offered a novel and encouraging new perspective that holds potential for further illuminating the clinicopathological relevance of PC as well as its molecular etiology.

## Introduction

Pancreatic cancer (PC) is ranked as the 14th most common cancer and the 7th highest cause of cancer mortality in the world^1,2^. According to projections provided by Globocan, there will be 458,918 new cases of PC diagnosed worldwide in 2018, and 432,242 people will lose their lives to the disease^3^. The age-standardized incidence is found to be at its peak in Europe and North America, while it is at its lowest in Africa and South Central Asia^4^. In general, affluent countries have greater incidence rates than developing countries do. This is a pattern that has been seen consistently. The most common cause of death and a key contributor to the potentially fatal nature of PC is a process called metastasis^5^. In clinical practice, the majority of patients are only detected at a very late stage, when metastatic spread is already present; at this time, the 5-year survival rate is only 3%^6^. Even in individuals who have undergone potentially curative surgical resection with clean tumor margins (R0), seventy-five percent of them would still succumb to recurrence and metastasis within 5 years following the procedure^7,8^. Because of the intricacy of the process, our understanding of the molecular processes that are involved in the metastatic cascade is yet only partially formed. PC is characterized by frequent occurrence of genetic and epigenetic abnormalities, which are connected with the improper activation of tumor driver genes. Thus, it is very important to locate new oncogenes that play a role in the carcinogenesis of PC and to enhance the overall survival rate of PC patients.

Research into cancers has had a consistent process of development over the course of the last few decades. In order for researchers to identify different forms of cancer before they exhibited any signs, they utilized a variety of techniques, such as screening at an earlier stage^9,10^. In addition to this, they have created novel approaches for the early prediction of the outcome of cancer therapy. Large volumes of information pertaining to cancer have been compiled as a result of the introduction of innovative technologies within the realm of medicine, and these findings are now at the disposal of the medical research community^11,12^. However, one of the most exciting and difficult tasks for doctors is trying to accurately forecast how a disease will progress in their patients. As a direct result of this, machine learning approaches have developed into a well-liked instrument for use in medical research^13^. These methods are able to find and detect patterns and links between them, from complicated datasets, while also having the ability to correctly forecast future outcomes of a particular form of cancer^14,15^.

In this study, we performed machine learning methods and identified three critical genes, including CTSE, LAMC2 and SLC6A14. A novel diagnostic model based on CTSE, LAMC2 and SLC6A14 showed a strong predictive ability in screening PC tissues from normal tissues in several GEO datasets. Then, we analyzed their prognostic value and confirmed SLC6A14 was distinctly related to poor prognosis of PC patients. Finally, our group carried out functional experiments to delve into the potential functions of SLC6A14 in PC cells. Overall, our research made significant strides in unraveling the intricate mechanisms that drive the pathogenesis and progression of PC. Our findings shed new light on the molecular pathways involved in the disease and have helped identify novel therapeutic targets for this deadly malignancy. Moreover, our work contributed to the discovery and validation of clinical biomarkers that can aid in the early detection and diagnosis of PC, thus improving patient outcomes.

## Methods

All methods were performed in accordance with relevant guidelines and regulations.

### Data acquisition

Five RNA-sequence files of PC were extracted from the GEO datasets, including GSE15471, GSE62452, GSE16515, GSE32676 and GSE28735. Using the databases Genotype-Tissue Expression (GTEx) and TCGA, we were able to gather sequencing data from both healthy and malignant pancreatic tissue. The clinical information of PC patients from TCGA datasets was shown in Table 1.

**Table 1:** Correlation of SLC6A14 expression with clinicopathological features of PC.

Characteristic	Low expression of SLC6A14	High expression of SLC6A14	*P*
n	89	89	
Gender, n (%)			0.097
Female	34 (19.1%)	46 (25.8%)	
Male	55 (30.9%)	43 (24.2%)	
Age, n (%)			0.072
< = 65	53 (29.8%)	40 (22.5%)	
> 65	36 (20.2%)	49 (27.5%)	
Pathologic stage, n (%)			0.320
Stage I	14 (8%)	7 (4%)	
Stage II	69 (39.4%)	77 (44%)	
Stage III	1 (0.6%)	2 (1.1%)	
Stage IV	3 (1.7%)	2 (1.1%)	
Histologic grade, n (%)			0.060
G1	21 (11.9%)	10 (5.7%)	
G2	44 (25%)	51 (29%)	
G3	21 (11.9%)	27 (15.3%)	
G4	2 (1.1%)	0 (0%)	
Age, mean ± SD	63.03 ± 10.2	66.46 ± 11.16	0.034

### Differentially expressed genes (DEGs) screening

Both the GSE62452 and GSE28735 datasets were merged into a single metadata cohort, and the combat function that is a part of the SVA package was applied to preprocess the data in order to get rid of any batch effects. With the assistance of the limma package of the R programming language, all of the background correction, normalization between arrays, and differential expression analysis that was done between 106 normal and 114 tumor samples were successfully completed. The cutoff thresholds for DEGs were chosen to be samples that met the requirements of having a lower corrected false discovery rate P and a log fold change (FC) that was greater than 2.

### Functional enrichment analysis

Using the “clusterProfiler” and DOSE packages in R, we ran enrichment studies for the Disease Ontology (DO), the Gene Ontology (GO), and the Kyoto Encyclopedia of Genes and Genomes (KEGG)^16^. We determined that words with a *p* value less than 0.05 and a q-value less than 0.05 were substantially enriched.

### Screening of the diagnostic genes

Analyses of LASSO can compress variable coefficients and cause certain regression coefficients to become zero by developing a penalty function. This helps achieve the goal of variable selection, which is to narrow the range of possible variables. The DEGs were used to perform the LASSO regression analysis in the “glmnet” package in R. This analysis was performed in order to screen the key genes. SVM-RFE has been applied in pattern recognition and classification problems. After that, the common genes that were found using the LASSO and SVM-RFE were analyzed by a ROC curve evaluation utilizing the “pROC” program. To be more precise, the validation of the genes was performed on the GSE15471, GSE16515, and GSE32676 datasets. In addition, we used the glm function to construct a binary classification Logistic model, meticulously specifying the model parameters, such as the family and link functions, to ensure its accuracy. Subsequently, we harnessed the capabilities of the rms package to meticulously craft a Nomogram model that is closely aligned with our Logistic model. The Nomogram model will not only provide valuable insights into the predictive power of our Logistic model but also allow us to visualize the relationship between the predictors and the outcome in a clear and interpretable manner.

### Analyzation of TIICs

The CIBERSORT analysis tool is a gene expression-based arithmetic that evaluates the data of immunocyte components from bulk cancer specimens by using a series of bar code genetic expression findings (a “signature matrix” of 547 genes). This “signature matrix” is comprised of the genes. Standardized genetic expression data sets were used and uploaded to the CIBERSORT online platform in order to achieve exact quantification of the proportion of 22 different immunocyte types present in PC samples. The number of permutations was set to 1000, and the quantity of possible outcomes was 22. Quantification was performed on a total of 22 different types of TIIC and metrics from CIBERSORT. The CIBERSORT p value was used to determine whether or not to eliminate the deconvolution that had a fit precision that was less exceptional. The p value provided by CIBERSORT allows one to identify their level of confidence in the results. Because p was less than 0.05, the analysis of the inferred fraction of the immunocyte subsets that was performed by CIBERSORT was considered to be trustworthy.

### Patients and clinical samples

Twelve paired PC and adjacent non-cancer specimens were collected from patients in our department. The patients had undergone surgery at Tianjin Fourth Central Hospital between 2021 and 2022. We have received approval from the Institutional Review Board of Tianjin Fourth Central Hospital before sample collection. The collected tissues were quickly stored in a − 80 °C freezer for the detection of expression. Informed consent was obtained from all patients and control subjects.

### PC cell lines and cell transfection

The human PC cell lines (BXpc-3, SW1990, MIApaca-2 and PANC-1) and human pancreatic duct epithelial (HPDE) cells that were employed in the investigation were obtained from the Type Culture Collection of the Chinese Academy of Sciences in Shanghai, China. During the course of the investigation, these cells were used. Every single one of the cells was cultured in DMEM media. All of these different media were supplemented with 10% fetal bovine serum (FBS), 100 units per milliliter (U/mL) of penicillin, and 100 ng/mL streptomycin. Both PANC-1 and MIApaca-2 cells were grown in 6-well plates at a density of 1 × 10^4^ cells per milliliter of medium. PANC-1 and MIApaca-2 cell lines were transfected with 50 nM shRNA-NC, Sh-SLC6A14-1 and Sh-SLC6A14-2 using Lipofectamine® 3000 (Invitrogen). This process was carried out following an incubation period of 24 h. pGPU6/Neo plasmid was cloned using short-hairpin RNA (sh-RNA) oligonucleotides and the associated negative controls in order to target SLC6A14. This plasmid was manufactured by GenePharma in Shanghai, China.

### RNA extraction and qRT-PCR analyses

The EASYspin Tissue and Cellular RNA Rapid Extraction Kit (Aidlab, Beijing, China) or the TRIzol Reagent (Invitrogen, USA) was used to extract total RNA from the PC cell lines and tissue samples, and the extraction process was carried out. After that, the concentration of total RNA was determined using a NanoDrop 2000 full-spectrum spectrophotometer (Thermo Scientific, USA). Following that, 1 μg of RNA was extracted from each sample and reverse-transcribed into cDNA in accordance with the instructions provided by the corresponding kit (Vazyme, Nanjing, China). Next, we carried out the qRT-PCR procedures in a 20 L reaction system utilizing a Bio-Rad IQ5 real-time PCR apparatus (Bio-Rad, USA) and a SYBR-Green PCR Master Mix (Vazyme, Nanjing, China). These instruments were manufactured in the United States. The sequences of the gene primers used in this investigation may be found in the supplementary Table S1.

### Cell proliferation assay

In order to determine whether or not PC cells are dividing, an experiment called the CCK-8 was carried out. To summarize, two times 103 cells were seeded into each well of the 96-well plate, and the plates were then cultured for 0, 24, 48, and 72 h, respectively. After that, 10ul of a CCK-8 solution that was manufactured by YEASENBio in Shanghai, China, was added, and the mixture was kept in the dark at 37 degrees Celsius for another two hours. The absorbance at 450 nm was measured in order to determine the number of cells that were still alive.

### Colony formation assay

In order to gain a deeper understanding of the cell proliferation process of PC cells, the cells were seeded in 6-well plates at a density of 5 × 10^2^ per well and then cultivated for 14 days. After fixing the colonies in ice-cold 70% methanol for ten minutes and staining them with crystal violet solution containing 0.5% for the same amount of time, we cleaned each well three times with PBS. The survival fraction (SF) were calculated as a ratio of the number of counted colonies and the number of the seeded colonies^17^.

### EdU assays

For the EdU tests, BeyoClick EdU-555 Kits manufactured in China by Beyotime were utilized. Cells were grown in a medium that contained 10 μM EdU prior to being fixed with 4% paraformaldehyde and then stained with EdU reaction buffer. The cells were stained with Hoechst so that the DNA could be seen, and the results were analyzed using a fluorescence microscope. Counts were taken of the cells that were positive for EdU.

### Migration and invasion assays

Cells that had been transfected were then collected for use in invasion and migration tests. Matrigel from Corning, USA, was first diluted with serum-free RPMI-1640 medium before being plated into the top chamber of an insert from Corning, USA, that had an 8-m pore size. This was done for the invasion assay. For the migration and invasion tests, a total of 5 × 10^4^ and 1 × 10^5^ cells were seeded into the top chamber, while medium containing 20% (FBS) was added to the bottom chamber. After a period of 36 h of incubation, the cells that were still present in the top chamber were removed, and the cells that were still in the bottom chamber were fixed with 4% paraformaldehyde and stained with crystal violet. Using a microscope (made by Olympus and located in Tokyo, Japan), researchers counted the amount of cells that moved to new locations or invaded existing ones.

### Western Blotting

The cells were collected and lysed with radioimmunoprecipitation assay (RIPA) lysis buffer (Beyotime, Shanghai, China) on ice for 30 min in both the experimental group and the control group. This was followed by ultrasonic oscillation at 20 W for 2 min and centrifugation at 12,000 *g* and 4 °C for 10 min. After collecting the supernatant, a measurement of the protein content was carried out. After that, 50 μg of proteins were separated using sodium dodecyl sulfate–polyacrylamide gel electrophoresis (spacer gel at 80 V for 40 min, and separation gel at 120 V for 90 min), deposited onto the membrane at 100 V for 90 min. At a temperature of 4 °C for twelve hours, the membranes were incubated with primary antibodies against SLC6A14, E-cadherin, N-cadherin, β-catenin, and GAPDH. The membrane was then washed a second time before the proteins were incubated with secondary antibodies at 37 degrees Celsius for one hour. After that, the membrane was washed, and then the picture was developed.

### Tumor growth assay in nude mice

Twelve male BALB/c nude mice (5-weeks of age) were bought from Shanghai SLAC corporation, and randomly divided into two groups. The following step was the infection of PANC-1 cells with either sh-SLC6A14 or sh-NC. The treated cells were then administered subcutaneously into the right flanks of mice at a rate of 1 × 10^7^ cells per animal. The mice were euthanized after a period of 28 days (4 weeks) during which the tumors were allowed to develop. Tumor and body weights were recorded, and tumor tissues were collected for further immunohistochemical analysis. The study was approved by the Institutional Research Ethics Committees of Affiliated Tumor Hospital of Tianjin Fourth Central Hospital. Mice were sacrificed by cervical dislocation. The study was reported in accordance with ARRIVE guidelines.

### Statistical analysis

Statistical analyses were performed using R software v3.5.0 (R Foundation for Statistical Computing, Vienna, Austria) and GraphPad Prism v7.00 (GraphPad Software Inc., USA). For the analysis of qualitative variables, we used either the Pearson's 2 test or Fisher's exact test. For the analysis of quantitative variables, we used either a t-test for paired samples or a non-parametric Wilcoxon rank-sum test for unpaired samples, depending on the circumstances. A one-way analysis of variance was performed on many different sets of normalized data. Chi-square analysis was used to determine whether or not there was an association between the level of SLC6A14 expression and each clinicopathological characteristic. The Kaplan–Meier method was used to map out overall survival curves, and the log-rank test was used to compare the results of the different groups. Cox proportional-hazard modeling was utilized for both the univariate and the multivariate analyses in order to evaluate the influence that factors play in survival. At the threshold of p less than 0.05, every difference was found to be statistically significant.

### Ethics approval and consent to participate

This research was approved by the Animal Ethics Committee of the First Affiliated Hospital of Tianjin Fourth Central Hospital and complied with The Guide for the Care and Use of Laboratory Animals.

## Results

### Identification of DEGs in PC and functional correlation analysis

**Figure 1:**
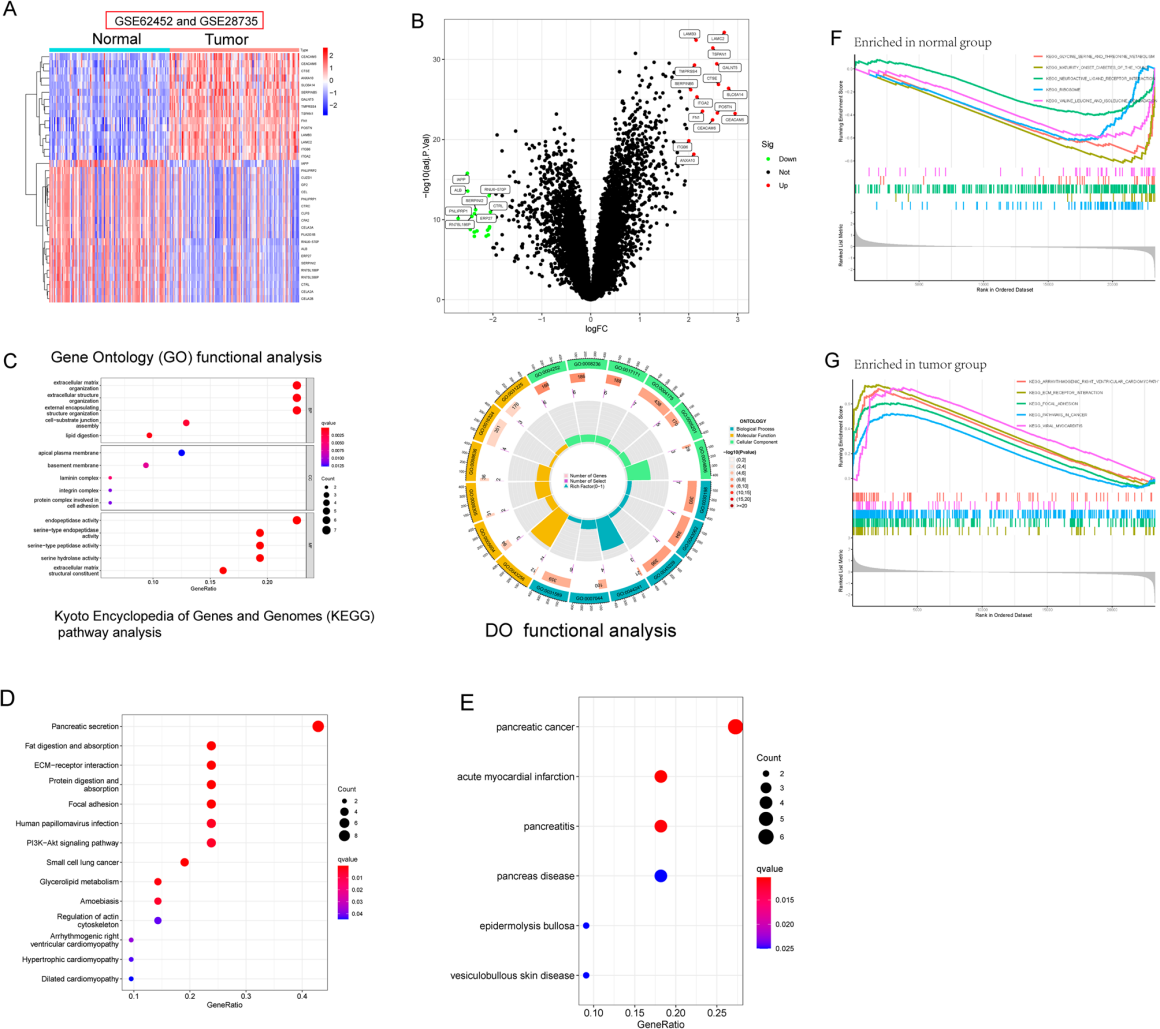
Identification of the DEGs in PC specimens and their enrichment analysis. (**A**,**B**) A total of 35 DEGs were shown in Heat Map and volcano plot based on GSE62452 and GSE28735 datasets. (**C**) The top 20 most significantly enriched BP, CC and MF terms. (**D**) The top 10 most significantly enriched KEGG pathways. (**E**) Disease ontology enrichment analysis of 35 DEGs. (**F**,**G**) Enrichment analyses via gene set enrichment analysis.

Data from two different GEO datasets (GSE62452 and GSE28735) were evaluated in a backwards fashion for the purpose of this study. After eliminating the impacts of batching, the limma software was used to do an analysis on the DEGs of the metadata. 35 DEGs were obtained: 15(Upregulation) and 20 genes (Downregulation) (Fig. 1A,B). Then, we performed GO analysis and found that 35 DEGs were mainly associated with extracellular matrix organization, extracellular structure organization, external encapsulating structure organization, apical plasma membrane, basement membrane, laminin complex, endopeptidase activity, serine-type endopeptidase activity and serine-type peptidase activity (Fig. 1C). The results of KEGG assays suggested that 35 DEGs were mainly enriched in Pancreatic secretion, Fat digestion and absorption, ECM-receptor interaction and PI3K-Akt signaling pathway (Fig. 1D). DO assays revealed that 35 DEGs were mainly related to PC, acute myocardial infarction, pancreatitis, pancreas disease, epidermolysis bullosa and vesiculobullous skin disease (Fig. 1E). The GSEA results demonstrated that the enriched pathways mainly involved in ARRHYTHMOGENIC_RIGHT_VENTRICULAR_CARDIOMYOPATHY, ECM_RECEPTOR_INTERACTION, FOCAL_ADHESION, PATHWAYS_IN_CANCER and IRAL_MYOCARDITIS (Fig. 1F, G).

### Identification and validation of diagnostic feature biomarkers

**Figure 2:**
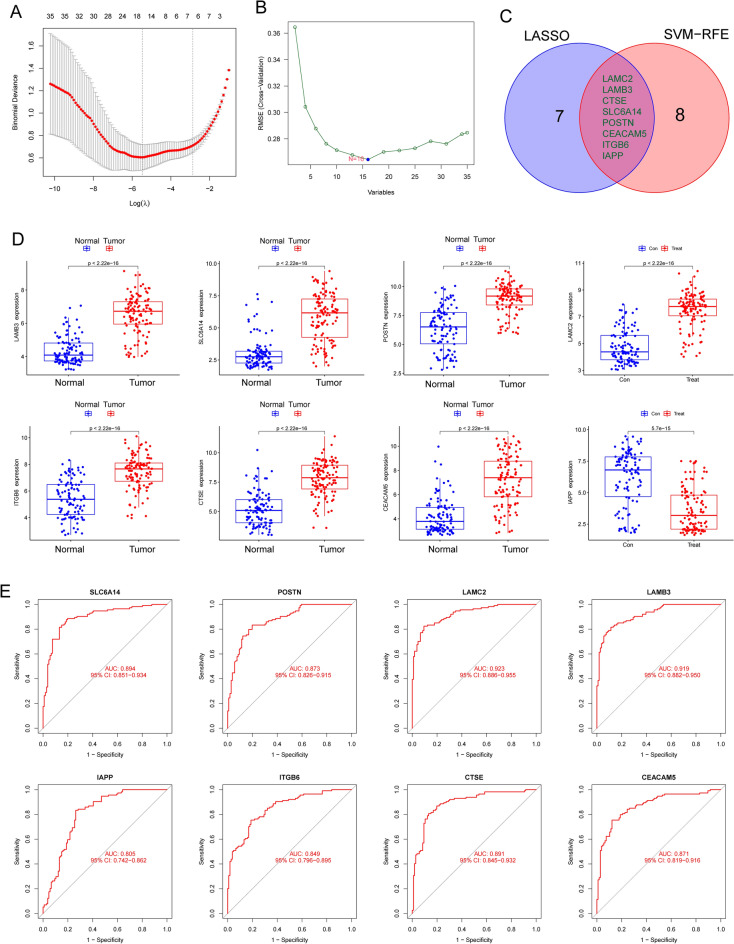
The process of screening potential diagnostic markers for the PC diagnosis. (**A**) The LASSO model’s tuning and feature selection process. (**B**) A graphic illustrating the biomarkers that were chosen using the SVM-RFE method. (**C**) Venn diagram demonstrating eight diagnostic markers shared by the above methods, including LAMC2, LAMB3, CTSE, SLC6A14, POSTN, CEACAM5, ITGB6 and IAPP. (**D**) The expression pattern of LAMC2, LAMB3, CTSE, SLC6A14, POSTN, CEACAM5, ITGB6 and IAPP in PC specimens and non-tumor specimens from GSE62452 and GSE28735 datasets. (**E**) ROC assays were applied to examine the diagnostic value of the above eight genes in screen PC specimens from non-tumor specimens.

**Figure 3:**
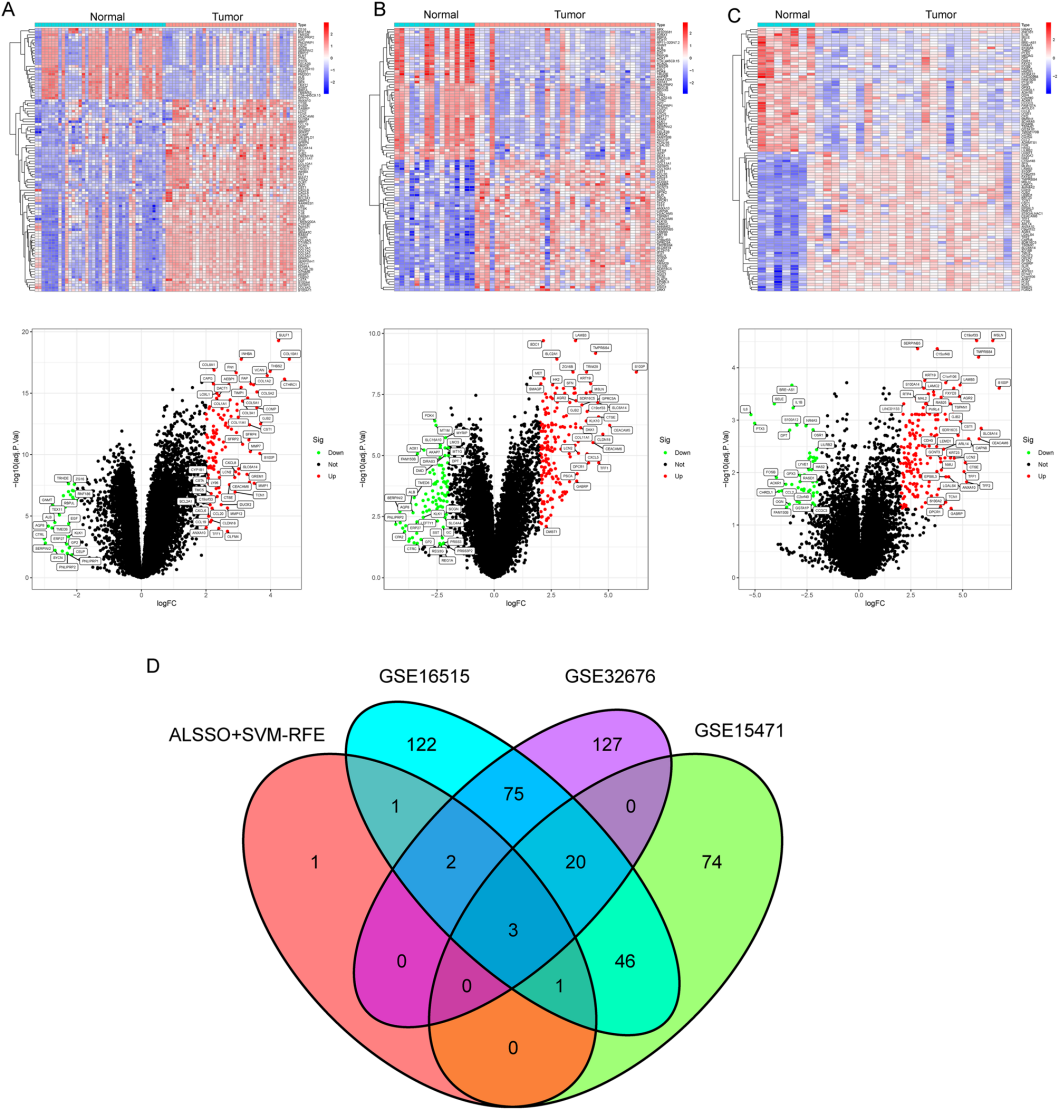
Identification of critical genes based on the machine learning and three GEO datasets. (**A**) 144 DEGs in PC specimens were identified using GSE15471 datasets. (**B**) 270 DEGs PC specimens were identified using GSE16515 datasets. (**C**) 227 DEGs were identified using GSE32676 datasets. (**D**) Venn diagram demonstrating three diagnostic markers shared by the machine learning and three GEO datasets, including LAMC2, CTSE and SLC6A14.

**Figure 4:**
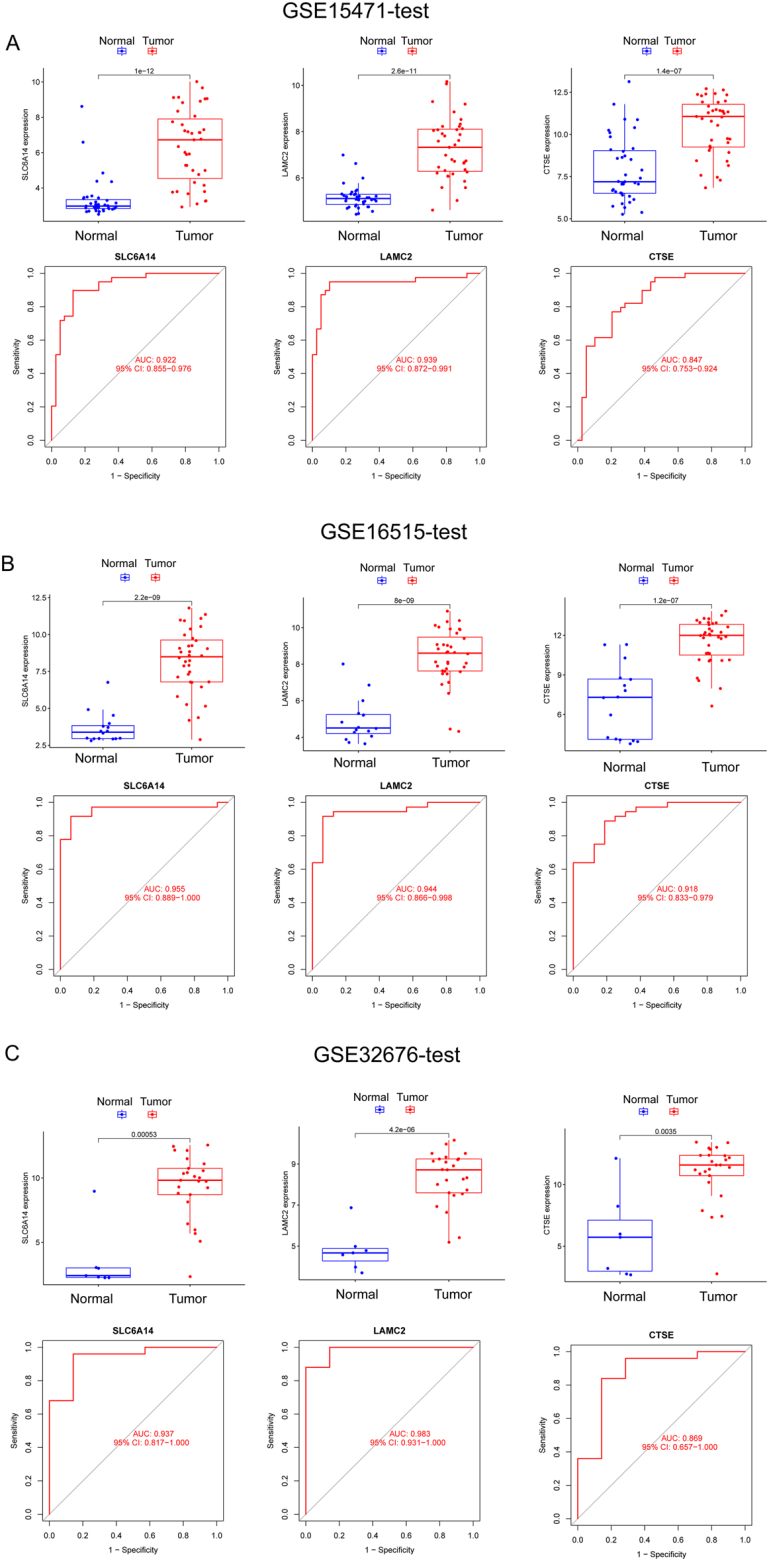
The expression and diagnostic value of LAMC2, CTSE and SLC6A14 were shown using (**A**) GSE15471 datasets, (**B**) GSE16515 datasets and (**C**) GSE32676 datasets.

**Figure 5:**
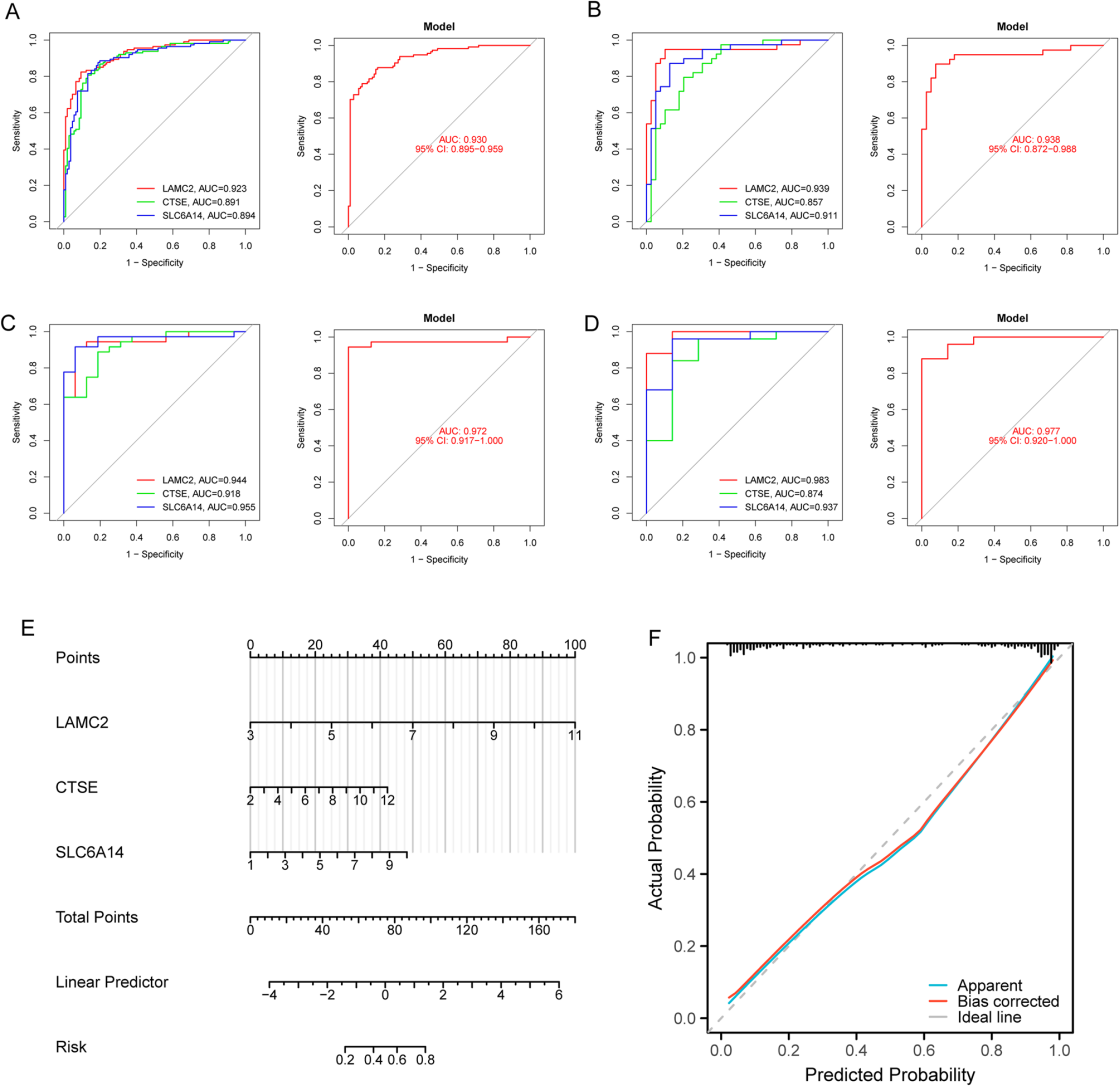
Logistic regression model to identify the AUC of disease samples from (**A**) GSE62452 and GSE28735 datasets, (**B**) GSE15471 datasets, (**C**) GSE16515 datasets and (**D**) GSE32676 datasets. (**E**) Nomogram to estimate the diagnostic value of three critical genes for PC. (**F**) Calibration plot for the diagnostic nomogram.

By the use of the LASSO regression technique, the DEGs were filtered down, and the outcome was the discovery of 15 variables as diagnostic biomarkers for PC (Fig. 2A). A subset of five features among the DEGs was determined using the SVM-RFE algorithm (Fig. 2B). The eight overlapping features (LAMC2, LAMB3, CTSE, SLC6A14, POSTN, CEACAM5, ITGB6 and IAPP) between these two algorithms were ultimately selected (Fig. 2C). The expression trend of eight overlapping features were shown in Fig. 2D. In addition, the results of ROC assays confirmed all eight genes showed a strong predictive ability in screening PC tissues from normal tissues (Fig. 2E). Then, we further explored the DEGs in PC using GSE15471, GSE16515 and GSE32676. 144 DEGs were identified in GSE15471 datasets (Fig. 3A). 270 DEGs were identified in GSE16515 datasets (Fig. 3B) and 227 DEGs were identified in GSE32676 datasets (Fig. 3C). Then, we screened three overlapping genes (LAMC2, CTSE and SLC6A14) between four datasets (Fig. 3D). The expressions of LAMC2, CTSE and SLC6A14 were distinctly increased in PC specimens in GSE15471, GSE16515 and GSE32676 datasets (Fig. 4A–C), and their diagnostic value was also demonstrated. Then, we developed a novel diagnostic model based on LAMC2, CTSE and SLC6A14. Importantly, the new model showed a strong predicted ability in merged datasets (Fig. 5A), GSE15471 (Fig. 5B), GSE16515 (Fig. 5C) and GSE32676 (Fig. 5D) datasets. Finally, we used the glm function to build a binary classification Logistic model and then performed the rms package to construct a related Nomogram model (Fig. 5E). In addition, by observing the calibration curve, the observed results were highly consistent with predicted results (Fig. 5F).

### The expression of LAMC2, CTSE and SLC6A14 in PC and its prognostic value based on TCGA datasets

**Figure 6:**
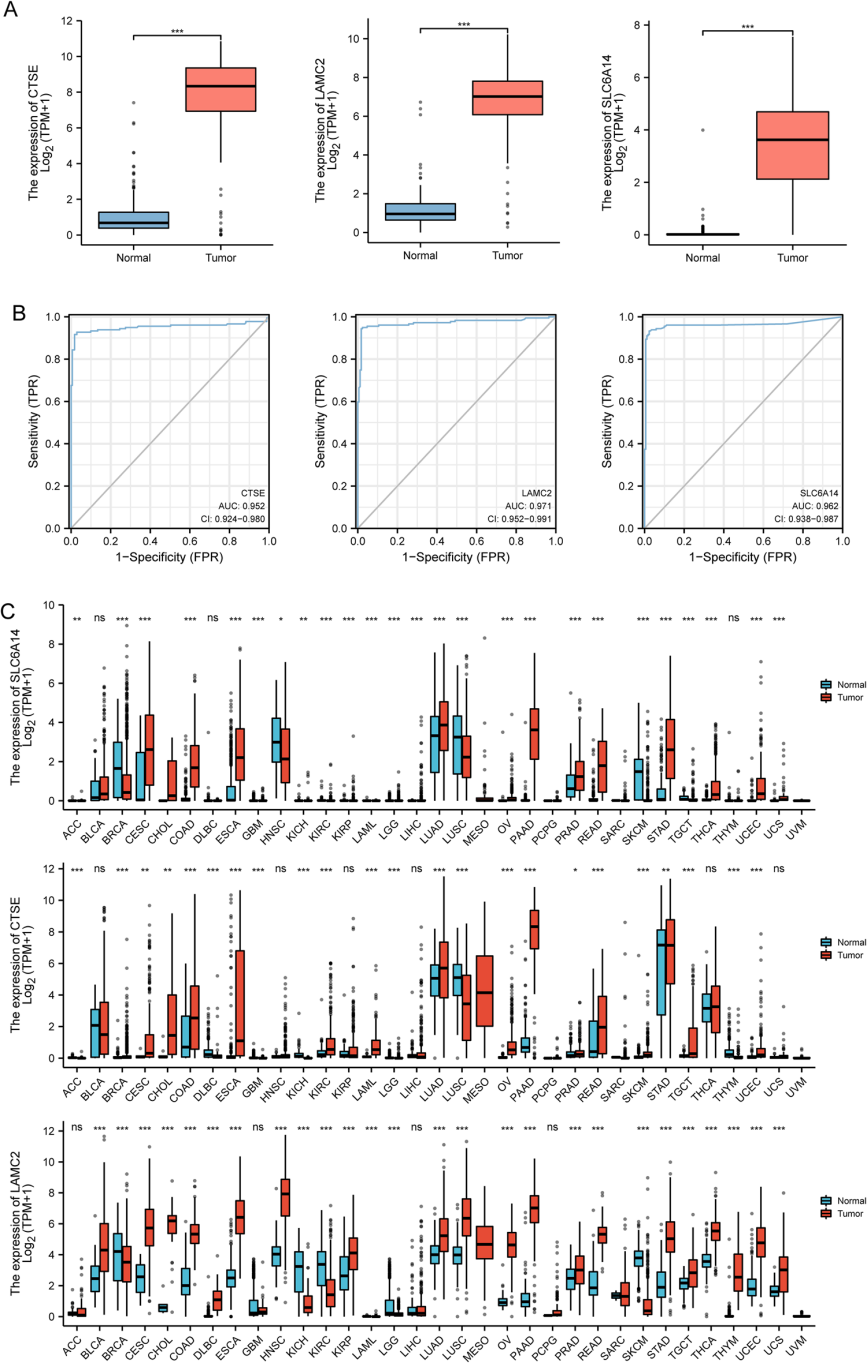
The expression of LAMC2, CTSE and SLC6A14 in TCGA datasets. (**A**) The expression of LAMC2, CTSE and SLC6A14 was distinctly increased in PC specimens compared with non-tumor specimens from TCGA datasets. (**B**) The diagnostic value of LAMC2, CTSE and SLC6A14 was confirmed. (**C**) Pan-cancer assays of LAMC2, CTSE and SLC6A1, which exhibited a dysregulated level in several types of tumors.

**Figure 7:**
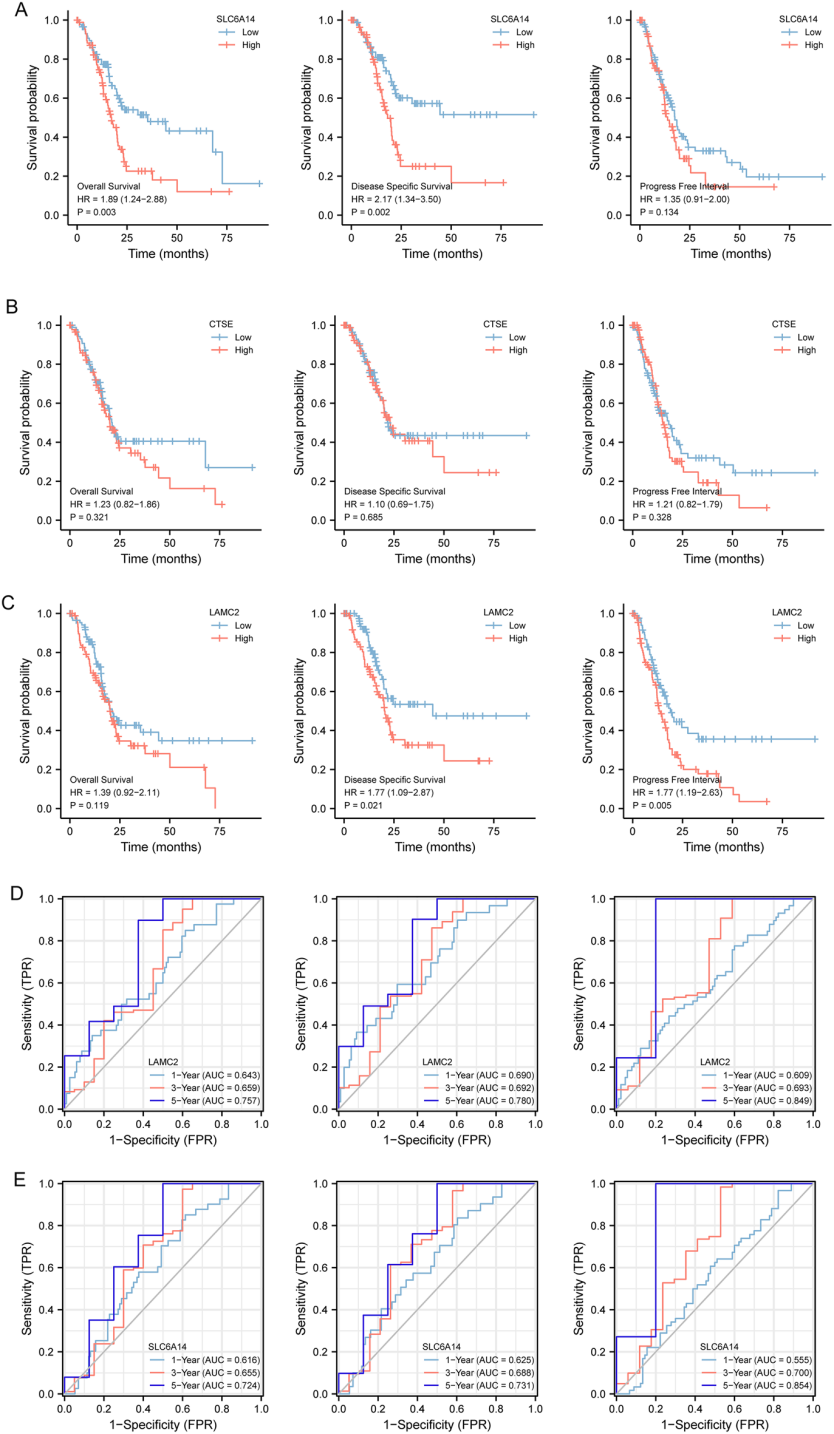
Kaplan–Meier survival curves comparing the high and low expression of LAMC2, CTSE and SLC6A14 in PC. (**A**) Survival curves of OS, DSS, and PFI between SLC6A14-high and -low patients with PC. (**B**) OS, DSS, and PFI survival curves for CTSE-high and -low patients with PC. (**C**) Compared OS, DSS, and PFI survival curves for LAMC2-high and -low patients with PC. (**D**,**E**) ROC analysis was used to evaluate the predictive power of LAMC2 and SLC6A14 for OS, DSS and PFI.

To further explore the expression of LAMC2, CTSE and SLC6A14 in PC, we analyzed TCGA datasets. As shown in Fig. 6A, we found that the expressions of LAMC2, CTSE and SLC6A14 was distinctly increased in PC specimens. Moreover, ROC assays also suggested the strong diagnostic value of LAMC2, CTSE and SLC6A14 for PC with AUC > 0.8(Fig. 6B). Moreover, we performed pan-cancer assays and found that LAMC2, CTSE and SLC6A14 exhibited a dysregulated level in many types of tumors, highlighting their potential function in tumor progression (Fig. 6C). Then, we further explored the prognostic value of LAMC2, CTSE and SLC6A14 in PC patients. Importantly, patients whose SLC6A14 expression was high had a lower OS and DSS than patients whose SLC6A14 expression was low (Fig. 7A). However, we did not observed the expression of LAMC2, CTSE was associated with OS of PC patients (Fig. 7B,C). In addition, we found high LAMC2 expression predicted a shorter DSS and PFI (Fig. 7C). Thus, we identified SLC6A14 and LAMC2 may be prognostic biomarkers for PC patients. The area under ROC curve (AUC) of SLC6A14 (Fig. 7D) and LAMC2 (Fig. 7E) expression based on TCGA data were > 0.7, suggesting that SLC6A14 had a strong predictive ability for the survival of PC patients. Univariate and multivariate Cox assays revealed that SLC6A14 expression was an independent prognostic factor for both OS (Table 2) and DSS (Table 3) of patients with PC (Table 4). In addition, we also found LAMC2 expression was an independent prognostic factor for both DSS (Table 5) and PFI (Table 6) of PC patients(Table 7).

**Table 2:** Prognostic factors for overall survival by univariate and multivariate analysis.

Characteristics	Total (N)	Univariate analysis		Multivariate analysis	
		Hazard ratio (95% CI)	*P* value	Hazard ratio (95% CI)	*P* value
Gender	178				
Female	80	Reference			
Male	98	0.809 (0.537–1.219)	0.311		
Age	178				
< = 65	93	Reference			
> 65	85	1.290 (0.854–1.948)	0.227		
Histologic grade	176				
G1&G2	126	Reference			
G3&G4	50	1.538 (0.996–2.376)	0.052	1.360 (0.872–2.122)	0.176
SLC6A14	178				
Low	89	Reference			
High	89	1.892 (1.244–2.879)	**0.003**	1.723 (1.121–2.649)	**0.013**

**Table 3:** Prognostic factors for disease specific survival by univariate and multivariate analysis.

Characteristics	Total (N)	Univariate analysis		Multivariate analysis	
		Hazard ratio (95% CI)	*P* value	Hazard ratio (95% CI)	*P* value
Gender	172				
Female	76	Reference			
Male	96	0.751 (0.473–1.194)	0.227		
Age	172				
< = 65	92	Reference			
> 65	80	1.067 (0.670–1.701)	0.784		
Histologic grade	170				
G1&G2	122	Reference			
G3&G4	48	1.616 (0.994–2.628)	0.053	1.400 (0.853–2.296)	0.183
SLC6A14	172				
Low	86	Reference			
High	86	2.171 (1.344–3.505)	0.002	2.004 (1.231–3.261)	0.005

**Table 4:** Prognostic factors for progress free interval by univariate and multivariate analysis.

Characteristics	Total (N)	Univariate analysis		Multivariate analysis	
		Hazard ratio (95% CI)	*P* value	Hazard ratio (95% CI)	*P* value
Gender	178				
Female	80	Reference			
Male	98	0.968 (0.658–1.423)	0.867		
Age	178				
< = 65	93	Reference			
> 65	85	1.256 (0.848–1.861)	0.256		
Histologic grade	176				
G1&G2	126	Reference			
G3&G4	50	1.684 (1.114–2.546)	**0.013**	1.684 (1.114–2.546)	0.013
SLC6A14	178				
Low	89	Reference			
High	89	1.349 (0.912–1.997)	0.134

**Table 5:** Prognostic factors for overall survival by univariate and multivariate analysis.

Characteristics	Total (N)	Univariate analysis		Multivariate analysis	
		Hazard ratio (95% CI)	*P* value	Hazard ratio (95% CI)	*P* value
Gender	178				
Female	80	Reference			
Male	98	0.809 (0.537–1.219)	0.311		
Age	178				
< = 65	93	Reference			
> 65	85	1.290 (0.854–1.948)	0.227		
Histologic grade	176				
G1&G2	126	Reference			
G3&G4	50	1.538 (0.996–2.376)	0.052	1.538 (0.996–2.376)	0.052
LAMC2	178				
Low	89	Reference			
High	89	1.391 (0.919–2.107)	0.119

**Table 6:** Prognostic factors for disease specific survival by univariate and multivariate analysis.

Characteristics	Total (N)	Univariate analysis		Multivariate analysis	
		Hazard ratio (95% CI)	*P* value	Hazard ratio (95% CI)	*P* value
Gender	172				
Female	76	Reference			
Male	96	0.751 (0.473–1.194)	0.227		
Age	172				
< = 65	92	Reference			
> 65	80	1.067 (0.670–1.701)	0.784		
Histologic grade	170				
G1&G2	122	Reference			
G3&G4	48	1.616 (0.994–2.628)	0.053	1.522 (0.933–2.482)	0.092
LAMC2	172				
Low	86	Reference			
High	86	1.769 (1.092–2.866)	**0.021**	1.651 (1.016–2.683)	**0.043**

**Table 7:** Prognostic factors for progress free interval by univariate and multivariate analysis.

Characteristics	Total (N)	Univariate analysis		Multivariate analysis	
		Hazard ratio (95% CI)	*P* value	Hazard ratio (95% CI)	*P* value
Gender	178				
Female	80	Reference			
Male	98	0.968 (0.658–1.423)	0.867		
Age	178				
< = 65	93	Reference			
> 65	85	1.256 (0.848–1.861)	0.256		
Histologic grade	176				
G1&G2	126	Reference			
G3&G4	50	1.684 (1.114–2.546)	0.013	1.552 (1.023–2.354)	0.039
LAMC2	178				
Low	89	Reference			
High	89	1.772 (1.192–2.633)	0.005	1.705 (1.140–2.551)	0.009

### Association between SLC6A14 and LAMC2 and immune cells

**Figure 8:**
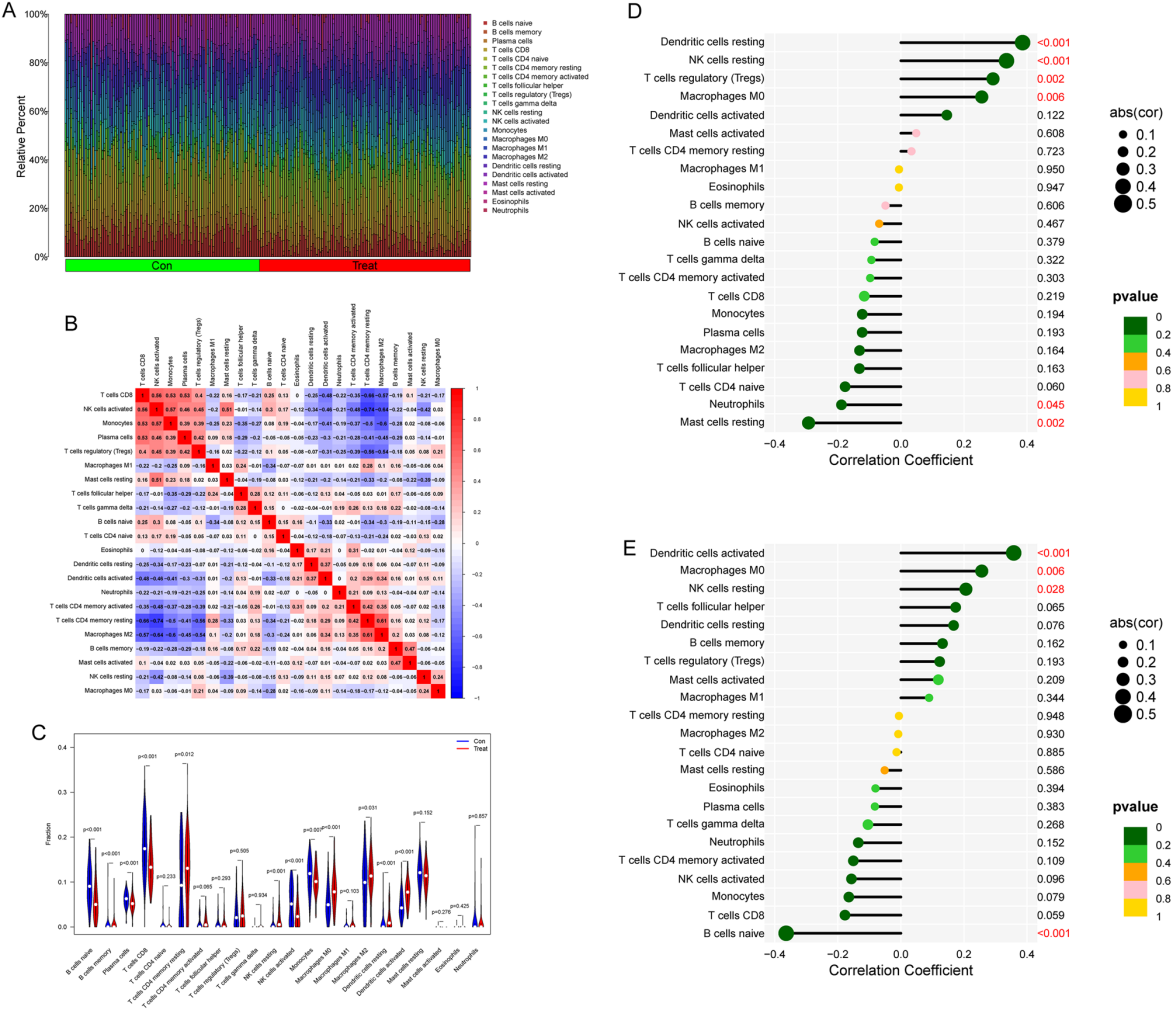
The association between SLC6A14, LAMC2, and infiltrating immune cells in PC patients. (**A**) Heatmap of 22 TIICs and immune cells among PC cases and normal specimens derived from an analysis of the GEO database. (**B**) In order to conduct an analysis of the matrix consisting of all 22 types of TIICs in PC, the Pearson correlation coefficient was utilized. (**C**) The unique composition of TIICs found in normal specimens as opposed to those found in tumor specimens is examined, along with the ramifications of this result. (**D**,**E**) Correlation between SLC6A14, LAMC2, and infiltrating immune cells in PC patients.

The composition of TIICs as well as the association between immune cells in PC specimens were displayed in Fig. 8A. In addition to this, we demonstrated the positive connections that exist between the various types of TIICs (Fig. 8B). In addition, we found significant differences in TIICs composition between normal and PC tissues (Fig. 8C). We found that the expression of LAMC2 was favorably linked with the expression of resting Dendritic cells, NK cells, T cells regulatory (Tregs), and Macrophages M0; however, a negative association was found between LAMC2 expression and resting Neutrophils and Mast cells (Fig. 8D). In addition, SLC6A14 expression was positively with Dendritic cells activated, Macrophages M0, NK cells resting, while negatively associated with B cells naïve (Fig. 8E).

### The identification of new diagnostic model in PC using TCGA datasets and our cohort

**Figure 9:**
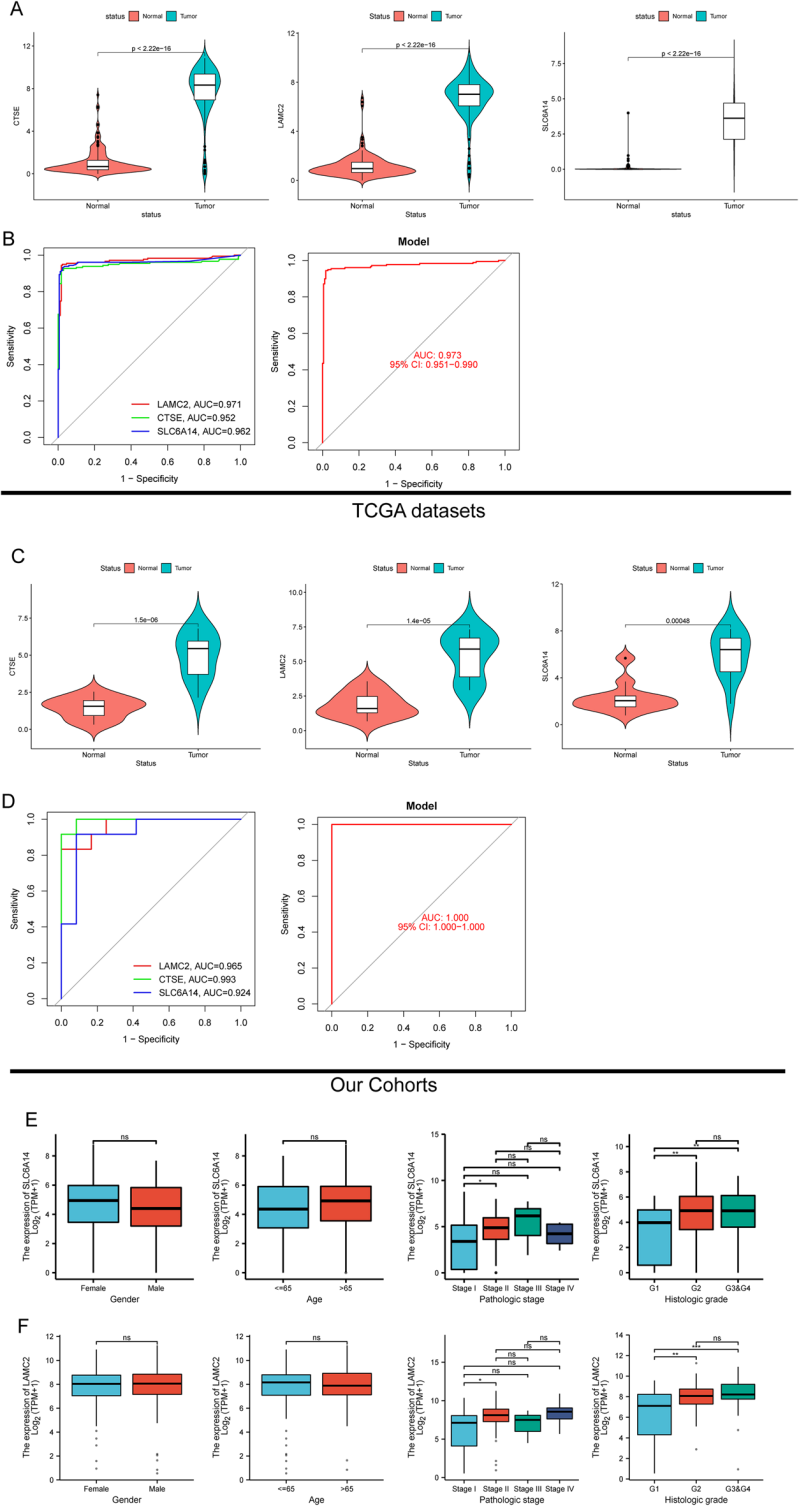
The expression and diagnostic value of new model was demonstrated in TCGA datasets and our cohort. (**A**) The expression of LAMC2, CTSE and SLC6A14 in TCGA datasets. (**B**) ROC for the diagnostic value of new model in TCGA datasets. (**C**) The expression of LAMC2, CTSE and SLC6A14 in our cohort. (**D**) ROC for the diagnostic value of new model in our cohort. (**E**,**F**)The association between SLC6A14 expression and LAMC2 expression and several clinical characteristics, including gender, age, pathologic stage and histologic grade.

To further demonstrate the diagnostic value of new diagnostic model in PC, we further analyzed TCGA datasets and found that the expression of LAMC2, CTSE and SLC6A14 were highly expressed in PC (Fig. 9A). The diagnostic model can be used to screen PC specimens from non-tumor specimens with AUC = 0.973 (Fig. 9B). Moreover, we also confirmed upregulation of LAMC2, CTSE and SLC6A14 in PC specimens from our cohort (Fig. 9C). In our cohort, the diagnostic value of new model was also demonstrated with AUC = 1.000 (Fig. 9D). The results of clinical assays revealed that both SLC6A14 (Fig. 9E) and LAMC2 (Fig. 9F) were associated with advanced pathologic stages and histologic grades.

### Knockdown of SLC6A14 suppressed the proliferation of PC cells in vitro and in vivo

**Figure 10:**
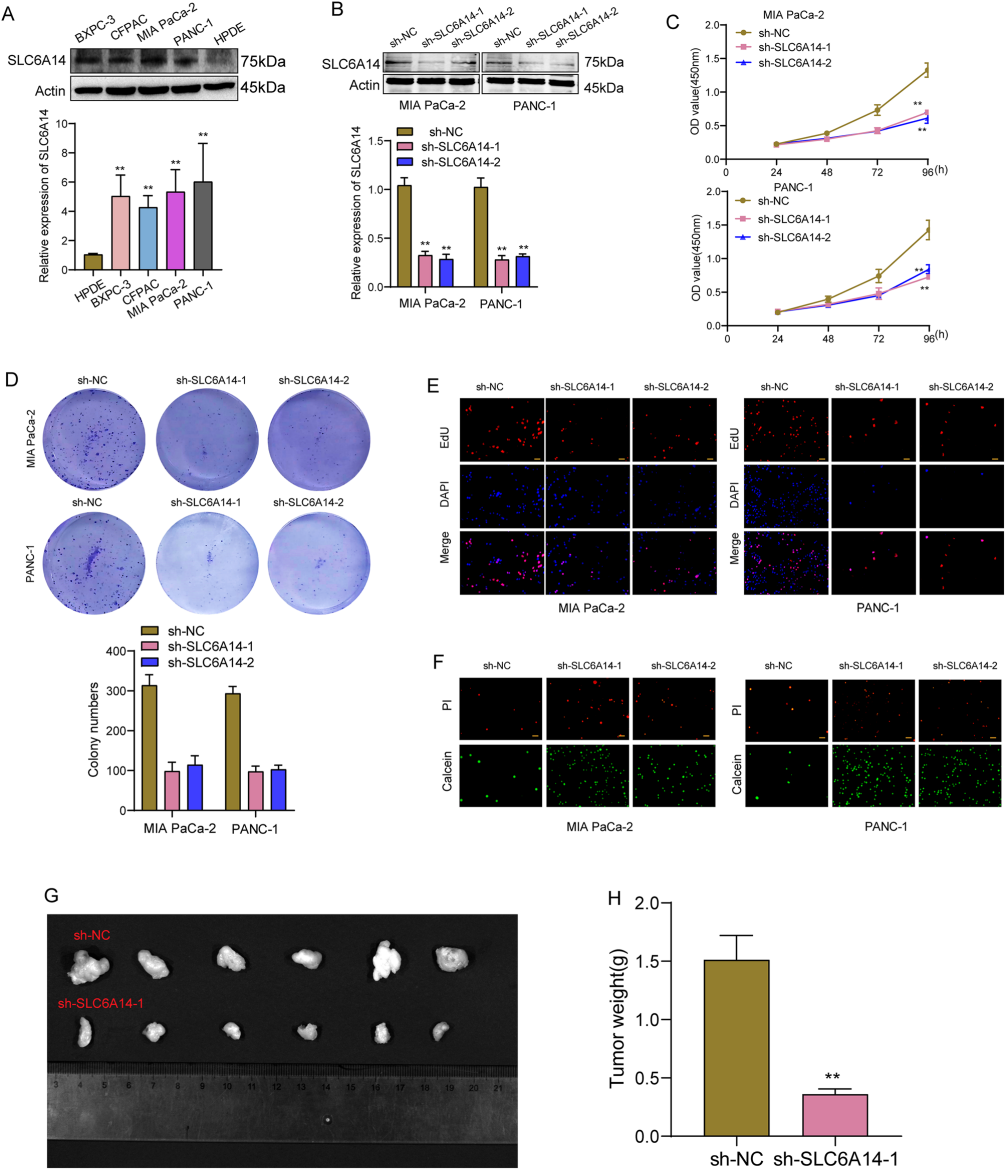
Knockdown of SLC6A14 suppressed the proliferation of PC cells in vitro and in vivo. (**A**) SLC6A14 expression was distinctly increased in four PC cell lines compared with HPDE cells. (**B**) RT-PCR confirmed the transfection efficiency of sh-SLC6A14-1 and SLC6A14-2 in PANC-1 and MIA paca-2 cells. (**C**) CCK-8 assays. (**D**) Clonogenic assays. (**E**) EdU assays. (**F**) Calcein-AM/PI assays. (**G**) Images of whole tumors that grew in nude mice after they were injected with PC cells that had been stably infected with sh-NC or sh-SLC6A14. (**H**) After the tumors had been removed from the various groups, the relative weights of each tumor were calculated.

Given that the clinical significance and function of SLC6A14 in PC was rarely reported, we further designed experiments to explore the potential function of SLC6A14 in PC progression. Firstly, we examined the expression of SLC6A14 in a few PC cells using western blot and RT-PCR and found that the level of SLC6A14 expression was noticeably higher in PC cells in comparison to HPDE cells (Fig. 10A). In addition, the transfection efficiency of shRNA targeting SLC6A14 in was further demonstrated by the use of RT-PCR and western blot (Fig. 10B). As shown in Fig. 10C–E, the findings of CCK-8 tests, EdU staining, and clonogenic assays all revealed that SLC6A14 knockdown significantly inhibited the proliferation of PANC-1 and MIApaca-2 cells. Moreover, Calcein-AM/PI assays revealed that SLC6A14 knockdown significantly increased PC cell apoptosis (Fig. 10F). To learn more about how SLC6A14 contributed to PC development, we conducted in vivo experiments and found that tumor weights in the SLC6A14-deficient group were significantly lower than those in the sh-control group (Fig. 10G, H).

### Knockdown of SLC6A14 suppressed metastasis of PC cells via Wnt/β‐catenin/EMT signalling pathway

**Figure 11:**
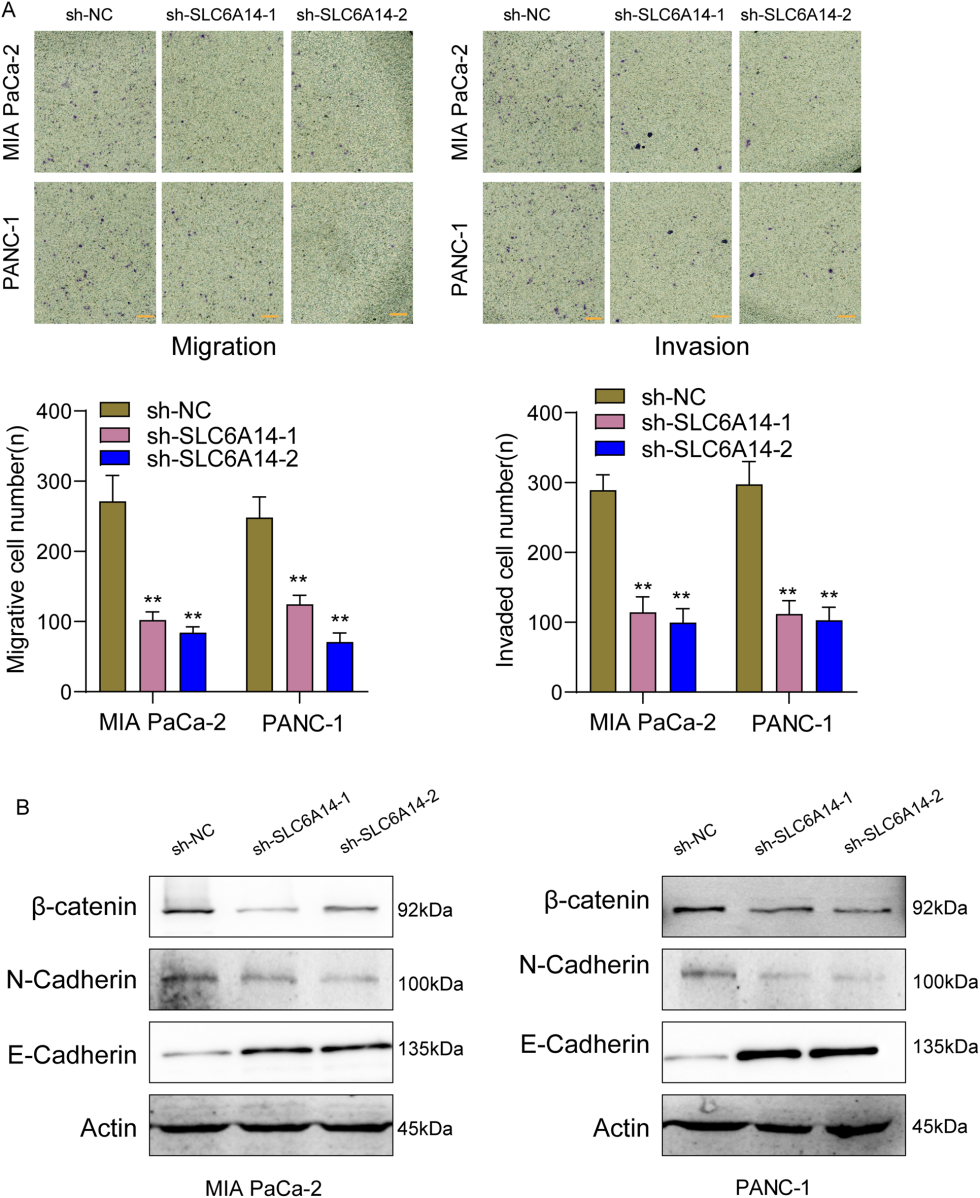
Knockdown of SLC6A14 suppressed metastasis of PC cells via Wnt/β‐catenin/EMT signalling pathway. (**A**) Transwell analysis was applied to examine the influence of SLC6A14 knockdown on the migration and invasion of PC cells. (**B**) The expression of β-catenin, E-cadherin and N-cadherin in both PANC-1 and MIApaca-2 cells after the transfection of sh-SLC6A14-1 and sh-SLC6A14-2.

Finally, we further explored whether SLC6A14 dysregulation may influence the metastasis of PC cells. As shown in Fig. 11A, we found that knockdown of SLC6A14 distinctly suppressed the migration and invasion of PANC-1 and MIApaca-2 cells. The Wnt/β-catenin/EMT signaling pathway is a complex pathway that plays important roles in embryonic development, tissue homeostasis, and cancer progression^18^. Its dysregulation can have significant pathological consequences, making it an attractive target for therapeutic intervention in certain diseases^19,20^. Importantly, we observed that knockdown of SLC6A14 distinctly suppressed the expression of β-catenin, E-cadherin and N-cadherin in both PANC-1 and MIApaca-2 cells (Fig. 11B).

## Discussion

Because of its high mortality rate, PC is categorized as a kind of gastrointestinal tumor that is universally thought to be terminal^21^. The only truly extreme treatment option available is surgical resection; nevertheless, the outlook is not good. There is no industry standard for the primary screening of high-risk variables for PC^22,23^. In contrast, imaging techniques like as computed tomography, magnetic resonance imaging, and positron emission tomography are used to diagnose PC^24^. However, the majority of people who have pancreatic cancer are already in advanced stages, and they have lost the chance to undergo surgical therapy after being detected^25–27^. With the rapid development of high-throughput sequencing technology, more and more tumor transcriptome data are being generated^28,29^. These data provide valuable information about the complex transcriptional regulatory networks of tumors^30^. However, due to the large scale and complexity of these data, traditional statistical analysis methods are no longer effective in analyzing them. Therefore, machine learning algorithms have become a popular method for analyzing these data. In this study, we performed LASSO and SVM-RFE using GSE62452 and GSE28735 datasets, and identified eight critical genes including LAMC2, LAMB3, CTSE, SLC6A14, POSTN, CEACAM5, ITGB6 and IAPP. They may be involved in PC progression. Then, we further analyzed GSE16515, GSE32676 and GSE15471 datasets, and confirmed LAMC2, SLC6A14 and CTSE exhibited a dysregulated level in all datasets. Then, we developed a novel diagnostic model using LAMC2, SLC6A14 and CTSE, which showed a strong predictive ability in screening PC specimens from non-tumor specimens in several GEO datasets. Our findings offer a novel diagnostic model for PC patients and highlighted the important function of LAMC2, SLC6A14 and CTSE in PC progression.

Given the diagnostic value of LAMC2, SLC6A14 and CTSE in PC patients, we further analyzed their expression and prognostic value using TCGA datasets. We found that the expression of LAMC2, SLC6A14 and CTSE was distinctly increased in PC specimens and exhibited a dysregulated level in many types of tumors, suggesting their important function in tumor progression. Interestingly, High levels of LAMC2 and SLC6A14 expression were the only ones we observed to correlate with PC patients' prognoses in the clinic. SLC6A14 expression was shown to be an independent prognosis factor for OS and DSS of PC patients, while LAMC2 expression was found to be an independent predictive factor for DSS and PFI, according to multivariate analysis. Previously, several studies have reported the function of LAMC2 and SLC6A14 in tumor progression. For instance, Zhang et al. reported that ovarian cancer tissues and cell lines showed an increased level of LAMC2 expression. The overexpression of LAMC2 led to an increase in the expression levels of p38, p-p38, c-myc, and CREB, as well as the nuclear translocation of the p38 protein. Additionally, the overexpression of LAMC2 greatly boosted cell proliferation and inhibited cell death. When the level of p38 in the cell was lowered, the effects of LAMC2 overexpression, which included the enhancement of cell proliferation and the suppression of cell death, were all significantly reduced. In addition, the expression of LAMC2 was suppressed by miR-125a-5p, which led to a reduction in the amount of p38 protein that accumulated in the nucleus. The effects of miR-125a-5p on cell proliferation inhibition, cell apoptosis promotion, and the suppression of tumorigenesis were considerably nullified by the upregulation of LAMC2, which also repressed tumorigenesis^31^. Liu et al. showed that the expression of LAMC2 was found to be significantly greater in NSCLC tissues compared to matching normal tissues. In vitro studies showed that inhibiting LAMC2 activity led to a reduction in NSCLC cell proliferation, migration, and invasion. The overexpression of LAMC2 was found to have a favorable association with the infiltration of macrophages into NSCLC tissues. The infiltration of THP-1 could be decreased by inhibiting the expression of LAMC2 in NSCLC cells, whereas the LAMC2 protein might increase the amount of THP-1 that is present. According to the findings of the Gene Set Enrichment Analysis, a high expression of LAMC2 is associated with focal adhesion and extracellular matrix receptor interaction^32^. Importantly, several studies have also confirmed LAMC2 expression was distinctly increased in PC specimens and predicted a poor prognosis of PC specimens, which was consistent with our findings^33^. On the other hand, the potential function of SLC6A14 in tumors was also reported. Mao et al. showed higher levels of SLC6A14 were connected with distant metastasis, advanced clinical stages and shorter survivals of CRC patients. SLC6A14 was shown to be significantly elevated in human CRC samples. Functionally, SLC6A14 substantially enhanced cell proliferation, prevented cell apoptosis, and worsened metastasis of CRC cells in vitro via enhancement of the JAK2/STAT3 Pathway. These effects were seen in CRC cells^34^. Furthermore, SLC6A14's oncogenic functions in gastric cancer and breast cancer have been documented^35,36^. Previously, a study reported that A pharmacological blockage of the SLC6A14 transporter interferes with amino acid feeding and decreases the development and proliferation of PC cells. SLC6A14 is highly up-regulated in PC. Based on these findings, SLC6A14 has been identified as a potential druggable target for PC. However, the clinical significance of SLC6A14 in PC was rarely reported. We provided evidence that SLC6A14 was highly expressed in PC in several GEO datasets, TCGA datasets and our cohort. Our findings suggested that SLC6A14 may be a novel diagnostic and prognostic biomarker for PC patients.

The tumor immune microenvironment refers to the microenvironment composed of immune cells, blood vessels, cytokines, and other factors around the tumor. It is an important component of tumor growth and development^37,38^. The tumor immune microenvironment contains various types of immune cells, such as T cells, B cells, macrophages, dendritic cells, and various types of cytokines and chemokines, such as tumor necrosis factor, interleukins, and chemokines^39^. These immune cells and factors can interact to form a network of immune reactions that regulate tumor growth and development^40^. In the tumor immune microenvironment, tumor cells can evade immune attacks through various mechanisms, such as inhibiting T cell activity, reducing antigen expression, and activating immune checkpoints. At the same time, the tumor immune microenvironment can also inhibit tumor growth and metastasis by activating immune cell responses^41,42^. In recent years, immunotherapy has become an important method of tumor treatment. By regulating immune cells and cytokines in the tumor immune microenvironment, immunotherapy can enhance tumor antigen expression and immunogenicity, thereby improving the possibility of tumor cell attack by the immune system and improving the efficacy of tumor treatment^43–45^. Our results demonstrated that the levels of SLC6A14 was positively associated with Macrophages M0 and NK cells resting. Studies have shown that in PC, an increase in the number of resting NK cells is associated with a poor prognosis. Specifically, resting NK cells are usually in a dormant state and do not have the ability to kill tumor cells, which may suppress the immune system's anti-tumor effects and thus affect the prognosis of PC patients. One study found that an increase in the number of resting NK cells was associated with shorter progression-free survival and overall survival in PC patients. In PC, an increase in M0 macrophages is associated with poor patient prognosis. Specifically, M0 macrophages can differentiate into M2 macrophages, which promote tumor cell growth and spread, thereby suppressing the immune system's anti-tumor effects and affecting the prognosis of PC patients^46^. According to the findings of one study, the presence of a greater number of M0 macrophages in patients diagnosed with PC was related with a shorter progression-free survival and overall survival^47^. Therefore, based on the findings of our study, immunosuppression brought on by an increased number of M0 macrophages cells in the primary tumor microenvironment may be the cause of a worse 5-year survival rate in patients with PC who have a high level of SLC6A14.

Our above results suggested SLC6A14 as a tumor promotor in PC progression. Then, we performed in vitro and in vivo experiments and found that knockdown of SLC6A14 distinctly suppressed the proliferation, migration, invasion and EMT pathway of PC cells, which confirmed our hypothesis. Tumor metastasis is one of the main factors contributing to poor prognosis in PC. PC often does not present obvious symptoms in its early stages, making it difficult to detect. By the time the tumor begins to spread to surrounding tissues or other organs in PC patients, it is often already in an advanced stage. At this point, treating the tumor becomes much more difficult, and the prognosis worsens accordingly. When PC cells enter the bloodstream or lymphatic system, they can metastasize to distant sites such as the liver, lungs, and peritoneum. This distant metastasis can significantly affect the prognosis of PC patients because it typically indicates a more severe disease state, which can weaken the effectiveness of treatment. Therefore, prevention of tumor metastasis and early treatment are crucial for PC patients. Thus, targeting the SLC6A14 gene may become a novel approach for treating PC.

Wnt/β-catenin signaling is a cellular signaling pathway and one of the important regulatory mechanisms for cell development and differentiation^48^. This pathway is regulated by the Wnt protein family and involves various cell types and tissues, including pancreatic tissue^49^. In normal conditions, the regulation of the Wnt/β-catenin signaling pathway is finely balanced and crucial for maintaining normal physiological functions. However, in certain situations, such as cancer development, the dysregulation of this signaling pathway can promote cell proliferation, invasion, and metastasis^50,51^. Therefore, the Wnt/β-catenin signaling pathway has become an important field for research and development of anticancer therapeutics. In recent years, several studies have reported that many tumor-related genes promoted PC progression via regulating Wnt/β-catenin signaling. In this study, we also found that knockdown of SLC6A14 distinctly suppressed the activity of Wnt/β-catenin signaling. However, further research is needed to understand how SLC6A14 specifically regulated Wnt/β-catenin signaling.

## Conclusion

We developed a novel diagnostic model based on LAMC2, SLC6A14 and CTSE in PC patients. SLC6A14 was substantially expressed in PC and was related with bad outcomes, immune infiltration and clinical progression of PC, all of which might possibly promote carcinogenesis through aberrant immune response. By regulating Wnt/β-catenin signaling, SLC6A14 has the potential to stimulate the proliferation, migration, and invasion of PC cells. This work offered a novel and encouraging new perspective that holds potential for further illuminating the clinicopathological relevance of PC as well as its molecular etiology.

### Supplementary Information

Supplementary Information.

## Data Availability

The datasets analyzed during the current study are available in The Cancer Genome Atlas database (TCGA, https://tcga-data.nci.nih.gov/tcga/) and the Gene Expression Omnibus database (GEO, https://www.ncbi.nlm.nih.gov/geo/) [GSE15471, GSE62452, GSE16515, GSE32676 and GSE28735]. The other data supporting the conclusions of this article will be made available by the authors, without undue reservation.
