# Supplementary material for: Machine learning identifies SLC6A14 as a novel biomarker promoting the proliferation and metastasis of pancreatic cancer via Wnt/β-catenin signaling
Source: Sci Rep. 2024 Jan 24;14:2116. doi: 10.1038/s41598-024-52646-8 (PMC10808089; doi:10.1038/s41598-024-52646-8)

Supplementary information

Supplemental Tables

Table S1 The primer sequences included in this study.

Name	primer sequences (5'–3')
SLC6A14: forward	ACCGTGGTAACTGGTCCAAAA
SLC6A14: reverse	CGCCTCCACCATTGCTGTAG
LAMC2: forward	GACAAACTGGTAATGGATTCCGC
LAMC2: reverse	TTCTCTGTGCCGGTAAAAGCC
CTSE: forward	AGGCATCCGTCCCTCAAGAA
CTSE: reverse	CCTTGGCACTCTGGTCCATTG
Actin: forward	CAGAGCCTCGCCTTTGCCGATCC
Actin: reverse	GACGACGAGCGCGGCGATATCA

Original Images for Figures

Figure 10

Figure 10A

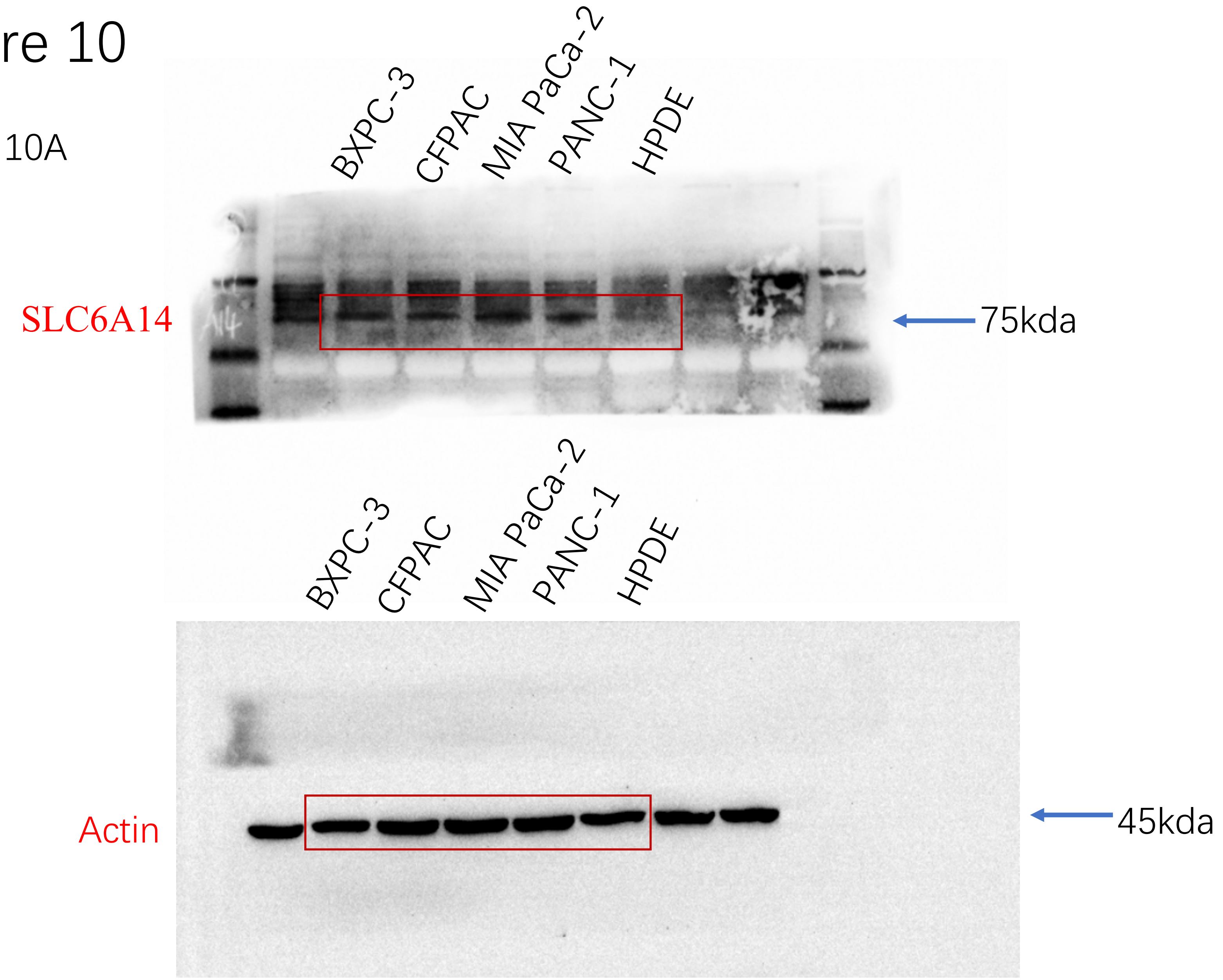

Figure 10B

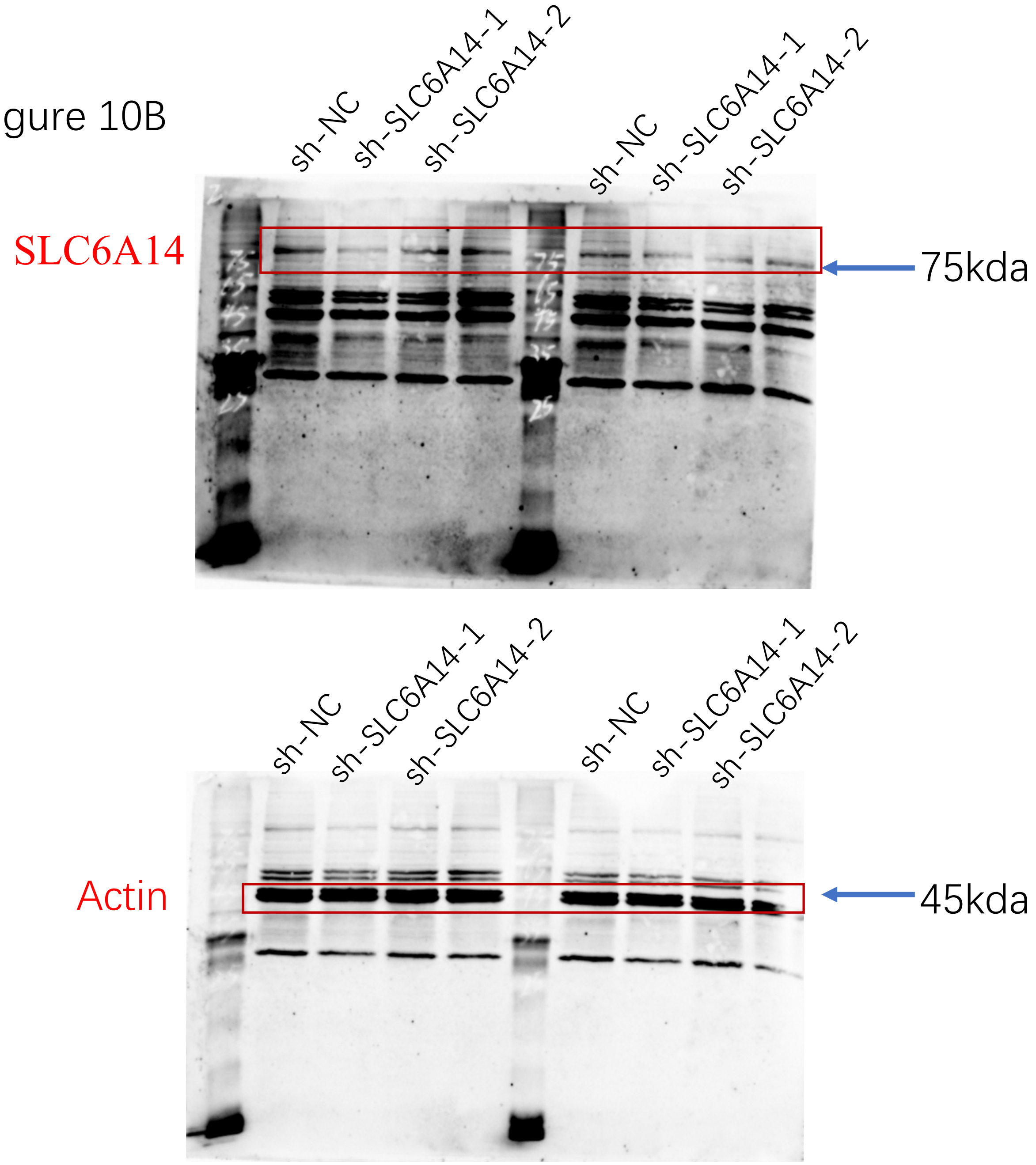
sh-NC
sh-SLC6A14-1
sh-SLC6A14-2
sh-NC
sh-SLC6A14-1
sh-SLC6A14-2
SLC6A14
75kda
sh-NC
sh-SLC6A14-1
sh-SLC6A14-2
sh-NC
sh-SLC6A14-1
sh-SLC6A14-2
Actin
45kda

Figure 11

Figure 11 B

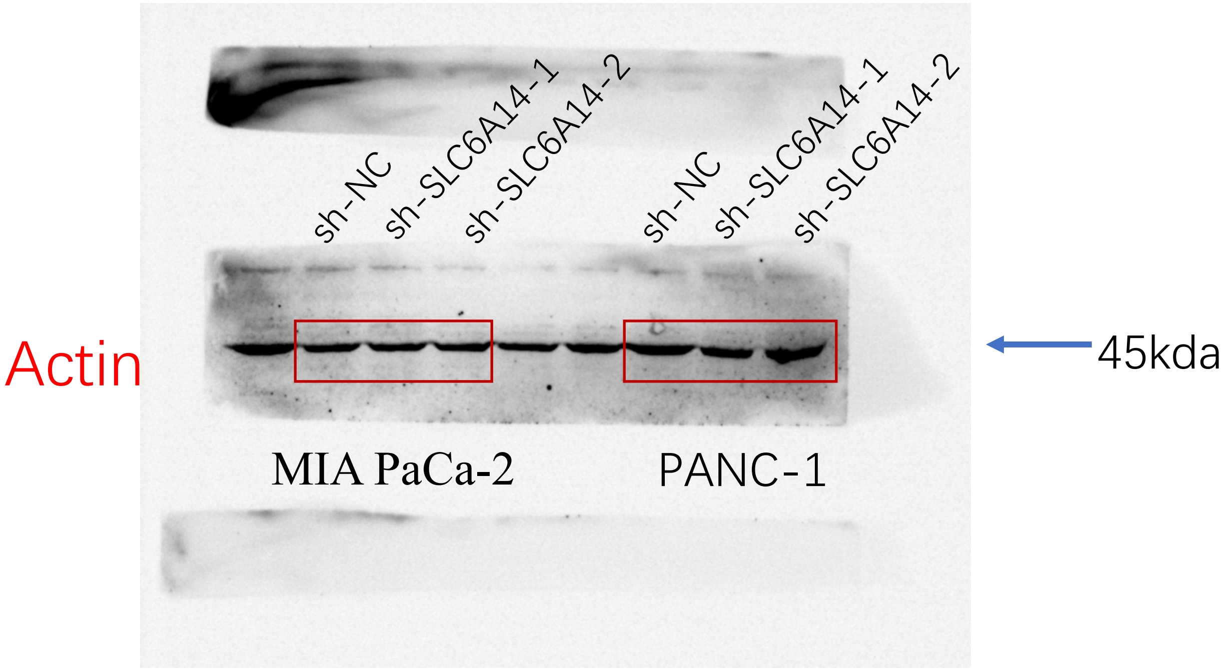

Figure 11 B

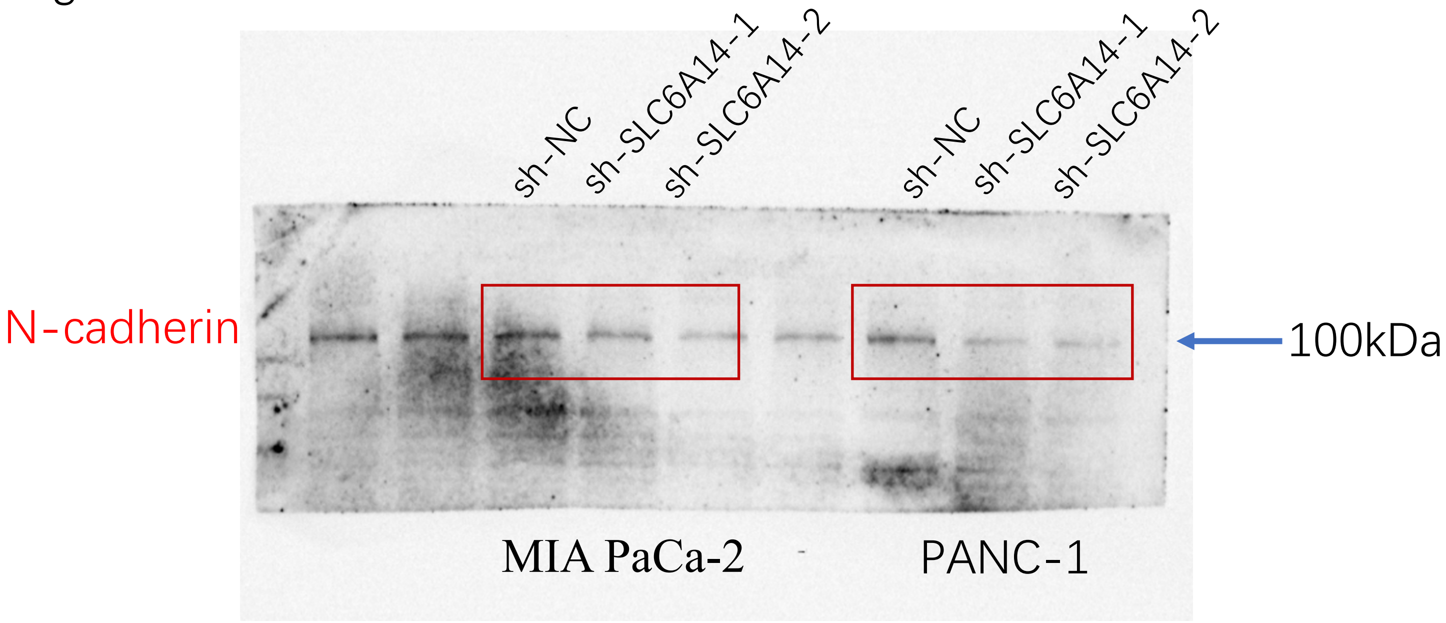

sh-NC
sh-SLC6A14-1
sh-SLC6A14-2
sh-NC
sh-SLC6A14-1
sh-SLC6A14-2
N-cadherin
100kDa
MIA PaCa-2
PANC-1

Figure 11 B

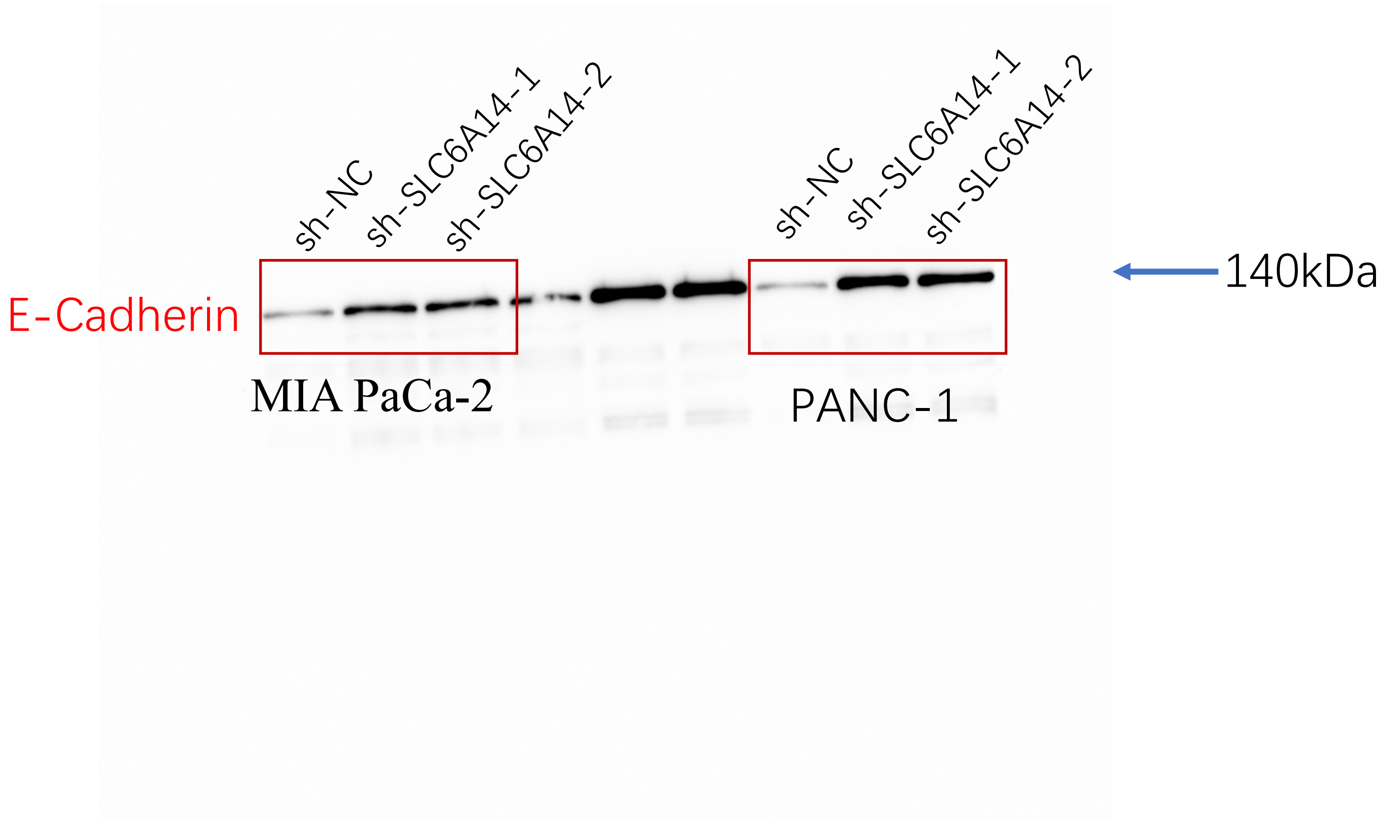

sh-NC
sh-SLC6A14-1
sh-SLC6A14-2
sh-NC
sh-SLC6A14-1
sh-SLC6A14-2
E-Cadherin
140kDa
MIA PaCa-2
PANC-1

Figure 11B

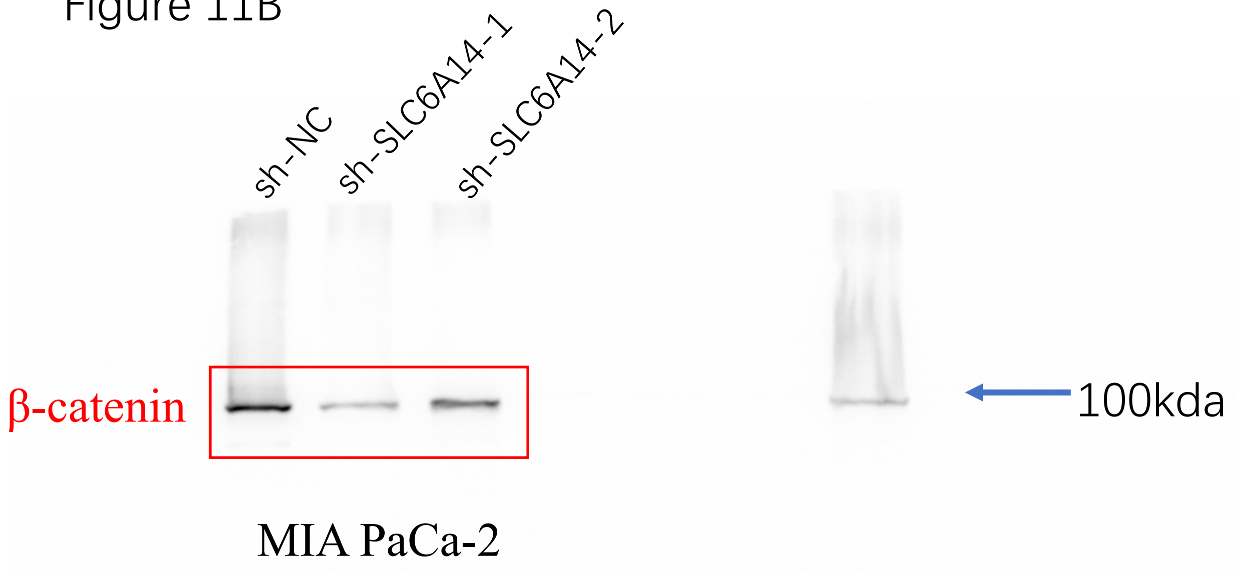

Figure 11B

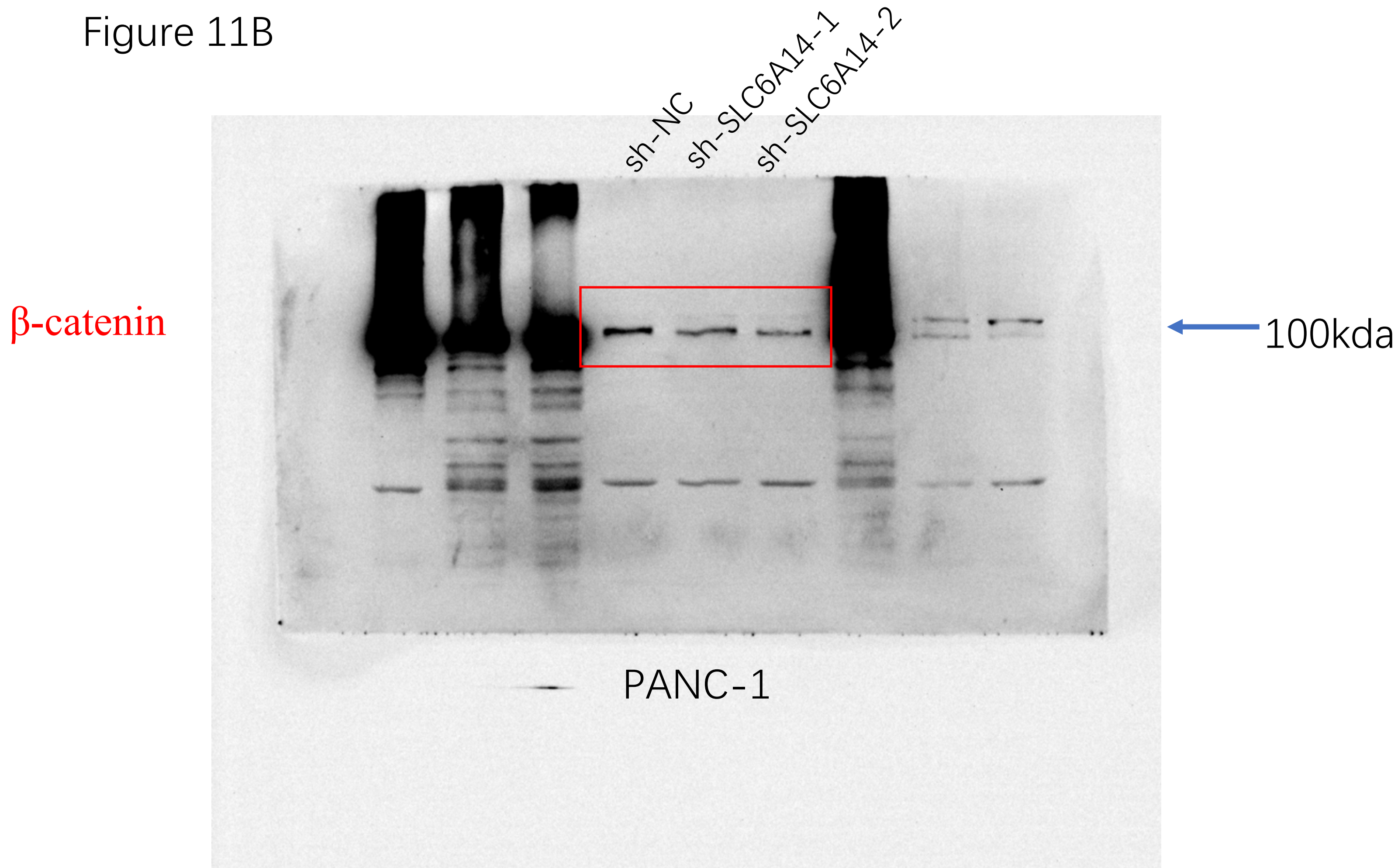

Supplement: Supplementary file 1 — Supplementary Information. [file 41598_2024_52646_MOESM1_ESM.pdf]
